# Reverse Mutation for Optimization Learning Artificial Lemming Algorithm and Its Application in Engineering

**DOI:** 10.3390/biomimetics11060389

**Published:** 2026-06-02

**Authors:** Mingbin Tang, Yejun Zheng, Lianbao Li, Li Cao, Zihao Cheng

**Affiliations:** 1 Engineering Technology Department, Shanghai Caoyang Vocational School, Shanghai 200333, China; tangmingbin71@163.com (M.T.); zhengyejun1983@gmail.com (Y.Z.); lilianbao98@163.com (L.L.); 2 School of Electronic and Electrical Engineering, Wenzhou University of Technology, Wenzhou 325035, China; 3 College of Control Science and Engineering, Zhejiang University, Hangzhou 310027, China; chengzihao@zju.edu.cn

**Keywords:** artificial lemming algorithm, Cauchy mutation, engineering optimization, improved salp swarm algorithm, reverse mutation for optimization learning

## Abstract

Complex engineering optimization problems often exhibit high-dimensional, multi-constraint, and nonlinear characteristics. Traditional deterministic optimization methods rely on gradient information and have limited optimization ranges, making it difficult to meet the requirements of efficient and accurate solutions. Intelligent optimization algorithms have become the core means of solving such problems. Aiming at the limitations of the standard artificial lemming algorithm (ALA), such as insufficient population diversity, premature convergence, weak local exploitation ability, and slow convergence speed, which make it difficult to meet the requirements of solving complex engineering optimization problems, this paper proposes a reverse mutation for optimization learning artificial lemming algorithm (RMALA). Based on the ALA algorithm, the algorithm integrates three strategies: Cauchy mutation, the improved salp swarm algorithm (ISSA), and reverse mutation for optimization learning. The Cauchy mutation is used to maintain population diversity and avoid premature convergence of the algorithm. The improved salp swarm algorithm enhances the local exploitation ability of the algorithm and improves the optimization accuracy. Reverse mutation for optimization learning guides the population toward the global optimal solution region and accelerates the convergence speed. The significant experimental results show that in the CEC2017 and CEC2022 standard test sets, as well as the three classic engineering constrained optimization problems of welded beams, cantilever beams, and pressure vessels, RMALA’s optimization accuracy is improved by more than 30% compared to the original ALA, and its convergence speed is improved by more than 25%. Its stability and robustness are better than those of five new swarm intelligence algorithms proposed in recent years. It can efficiently solve complex high-dimensional, nonlinear constrained optimization problems and has high significant engineering application value and academic innovation.

## 1. Introduction

In modern engineering design, intelligent control, data analysis, and other fields, complex optimization problems are increasing rapidly. Most of these problems exhibit high dimensionality, multiple constraints, strong nonlinearity, and a dense distribution of local optima [1,2]. Traditional deterministic optimization methods rely on gradient information of the objective model and possess a limited search range [3]. When solving such problems, these methods frequently suffer from low optimization efficiency, insufficient accuracy, and a high risk of premature convergence to local optima, making it difficult to meet the high-efficiency solution requirements of engineering applications [4]. Intelligent optimization algorithms are inspired by the swarm intelligence behaviors of biological groups in nature. By simulating behaviors such as foraging, migration, and cooperation, these algorithms realize global exploration and local exploitation of the solution space [5,6]. With the advantages of simple structure, strong robustness, gradient-free mechanism, and wide adaptability, they have become the core technique for addressing complex optimization problems and have been widely applied in mechanical design, civil engineering, aerospace, and many other fields [7]. In recent years, domestic and international scholars have proposed a series of classic intelligent optimization algorithms, including the Genetic Algorithm [8], Particle Swarm Optimization [9], Ant Colony Algorithm [10], and salp swarm algorithm [11], which provide effective solutions for complex optimization problems. However, with the continuous growth of problem complexity, traditional intelligent optimization algorithms have gradually exposed critical drawbacks. The Genetic Algorithm tends to converge prematurely and exhibits slow convergence in the later stage. Particle Swarm Optimization (PSO) lacks sufficient local exploitation ability in high-dimensional problems and easily falls into local optima. The Ant Colony Algorithm suffers from high parameter sensitivity and low optimization accuracy [12,13,14]. To overcome these limitations, researchers have continuously explored strategies for algorithm improvement and hybridization [15,16]. By introducing mutation mechanisms, opposition-based learning strategies, algorithm fusion, and other techniques, new hybrid optimization algorithms have been constructed to balance global exploration and local exploitation, thereby enhancing overall optimization performance [17,18].

The artificial lemming algorithm (ALA) is a newly developed intelligent optimization algorithm. It simulates population behaviors of lemmings in nature, such as foraging, migration, and burrowing, and establishes simple population update rules [19,20]. It features few parameters, easy implementation, and low computational complexity, and has shown certain optimization feasibility in low-dimensional and simple optimization problems [21,22]. Nevertheless, when applied to high-dimensional, complex, and multi-constrained engineering optimization problems, the limitations of the standard ALA become prominent [23]. It lacks effective population diversity maintenance. The foraging behavior that centers on the optimal individual easily causes population aggregation. The fixed randomness of migration behavior restricts its ability to avoid premature convergence. Its local exploitation capability is weak because core parameters are fixed and cannot be dynamically adjusted during iteration, making it difficult to approach the global optimum efficiently. Moreover, the lack of coordination between global exploration and local exploitation leads to slow convergence and low optimization accuracy. ALA also shows poor stability, as it is highly affected by initial population and random perturbations, resulting in large dispersion among multiple runs, which hinders the provision of reliable engineering optimization solutions [24,25,26]. These drawbacks have severely restricted the further application and popularization of ALA in engineering practice. Therefore, improving ALA to enhance its comprehensive optimization performance is of great theoretical significance and engineering application value [27].

Based on this, aiming at the limitations of the standard ALA, this paper integrates three strategies: Cauchy mutation, an improved salp swarm algorithm, and opposition-based mutation for optimization learning, and proposes a reverse mutation for optimization learning artificial lemming algorithm (RMALA). This paper first elaborates on the basic principles and limitations of the standard ALA, then details the design ideas and implementation methods of each improvement strategy, and constructs the complete algorithm flow of the RMALA. Subsequently, through benchmark test function experiments, the performance of the RMALA is compared with that of the standard ALA and other mainstream intelligent optimization algorithms to verify the optimization accuracy, convergence speed, and stability of the algorithm. Finally, the RMALA is applied to typical engineering constrained optimization cases to verify its engineering practicability. The research findings of this paper not only enrich the improvement theory of intelligent optimization algorithms but also provide an efficient and reliable new method for solving complex engineering optimization problems, which is of great significance for promoting the application and development of intelligent optimization algorithms in the engineering field.

Aiming at the shortcomings of the standard artificial lemming algorithm (ALA), such as insufficient population diversity, easy premature convergence, weak local exploitation ability, and slow convergence speed, this paper proposes a reverse mutation for optimization learning artificial lemming algorithm (RMALA) by integrating three strategies: Cauchy mutation, the improved salp swarm algorithm, and reverse mutation for optimization learning. The main contributions of this paper are as follows:

(1) Cauchy mutation is introduced into the long-distance migration behavior of the artificial lemming algorithm. The adaptive disturbance coefficient dynamically adjusts the disturbance intensity, which effectively maintains population diversity, alleviates the premature convergence of the algorithm, and improves the global exploration ability.
(2) The burrowing behavior is reconstructed by using the improved salp leader strategy, and the optimal solution guidance mechanism is introduced to strengthen the local exploitation ability of the algorithm and improve the optimization accuracy and convergence efficiency.
(3) A reverse mutation for the optimization learning strategy is proposed to improve the foraging behavior. By generating opposite solutions and performing optimal mutation, the population is guided to search rapidly towards the optimal solution region, balancing the global exploration and local exploitation process.
(4) Through the tests of CEC2017 and CEC2022 benchmark functions and three kinds of engineering constrained optimization examples (welded beam, cantilever beam, and pressure vessel), the comprehensive advantages of the RMALA in optimization accuracy, convergence speed, stability, and robustness are fully proved, verifying the practicability and reliability of the algorithm in solving complex engineering optimization problems.

## 2. Artificial Lemming Algorithm and Its Limitations

### 2.1. Basic Principles of Artificial Lemming Algorithm

The ALA simulates the swarm behaviors of lemmings in nature, regards each lemming individual as a solution vector in the search space, and realizes global search and local exploitation through three core behaviors [28,29,30] and finds the global optimal solution through population iteration. The artificial lemming algorithm mainly draws inspiration from four behaviors of lemmings: long-distance migration, burrowing, foraging, and avoiding predators [31,32,33].

(1) Initialization stage

The ALA generates the initial population by randomization, and the position of each lemming corresponds to a solution of the optimization problem. For a dim-dimensional optimization problem with a lemming population of size *N*, the candidate solution set of lemmings is an *N* × *dim* matrix, as shown in Formula (1).

(1) $$X=\left(\begin{array}{ccc} x_{1,1} & \ldots & x_{1,dim}\\ \vdots & \ddots & \vdots \\ x_{N,1} & \ldots & x_{N,dim} \end{array}\right)$$

The population initialization formula for the ALA is as follows:

(2) $$X=lb_{j}+rand\times \left(ub_{j}-lb_{j}\right)$$

where *rand* is a random value in the range of 0–1, and $lb_{j}$ and $ub_{j}$ represent the lower and upper bounds of the *j*-th dimension of the optimization problem, respectively.

(2) Long-distance migration behavior

When the number of lemmings is excessive, food shortage will occur. By finding habitats with abundant food resources, lemmings can obtain better living conditions and resources. The migration behavior of lemmings is affected by the positions of the optimal lemmings and random individuals in the population. The formula for its long-distance migration behavior mode is as follows:

(3) $$X_{i}(t+1)=X_{best}(t)+F\;\times BM\times \;\left(R\times \left(X_{best}(t)-X_{i}(t)\right)+(1-R)\times \left(X_{i}(t)-X_{a}(t)\right)\right)$$

(4) $$f_{BM}(x;0,\;1)=\frac{1}{\sqrt{2\pi }}\times exp(-\frac{x^{2}}{2})$$

(5) $$F=\left\{\begin{array}{c} 1,\;\;if[2\times rand+1]=1\\ -1,\;if[2\times rand+1]=2 \end{array}\right.$$

(6) $$R=2\times rand\left(1,\;dim\right)-1$$

where $X_{i}\left(t\right)$ and $X_{i}\left(t+1\right)$ represent the positions of the *i*-th lemming in the *t*-th and (*t* + 1)-th iterations, respectively. $X_{best}$ represents the current optimal solution. $X_{a}$ represents a randomly selected search individual in the population, and a is a random integer between 1 and *N*. *F* is a random search direction with a value of 1 or −1, and *BM* is the Brownian motion random walk coefficient expressed by Formula (4) [34,35,36]. *R* is a random number in the interval (−1, 1).

(3) Burrowing behavior

Lemmings will dig new burrows according to the position of existing burrows and the position of random individuals in the population. This behavior not only improves the efficiency of lemmings in finding food, but also enhances the ability to avoid predators and increases the survival probability of the population [34]:

(7) $$X_{i}(t+1)=X_{i}(t)+F\times L\times (X_{best}(t)-X_{b}(t))$$

(8) $$L=rand\times \left(1+sin\left(\frac{t}{2}\right)\right)$$

where *L* is a random number related to the current iteration number *t*. $X_{b}$ represents a randomly selected search individual in the population, and *b* is a random integer between 1 and *N*.

(4) Foraging behavior

Lemmings move near burrows in their habitats, and their foraging behavior mainly relies on an acute sense of smell and hearing to find food. In order to ingest as much food as possible, lemmings will forage in a spiral search mode in the foraging area, and the specific formula is as follows:

(9) $$X_{i}(t+1)=X_{best}(t)+F\times spiral\;\times rand\;\times X_{i}(t)$$

where spiral represents the spiral shape of the random search in the foraging process, which is calculated by Formulas (10) and (11):

(10) spiral=radius×(sin(2 ×π ×rand)+cos(2 ×π×rand))

(11) $$radius=\sqrt{\sum_{j=1}^{dim}{\left(X_{best,j}(t)-X_{i,j}(t)\right)}^{2}}$$

The radius in the spiral shape formula is the *radius* of the foraging range, that is, the Euclidean distance between the current position and the optimal solution.

(5) Predator avoidance behavior

When lemmings encounter danger, they will use their special running ability to flee back to their burrows and make deceptive movements to escape the pursuit of predators. The behavior mode of lemmings avoiding predators is as follows:

(12) $$X_{i}(t+1)=X_{best}(t)+F\;\times G\;\times Levy(dim)\times \left(X_{best}(t)-X_{i}(t)\right)$$

(13) $$G=2\times \left(1-\frac{t}{T_{max}}\right)$$

where *G* is the escape coefficient, indicating that their escape ability decreases with the increase in the iteration number. *T_max_* represents the maximum number of iterations. *Levy*(*dim*) is the Levy flight function to simulate the deceptive behavior of lemmings in the escape process [37,38,39]. The formula for the Levy flight function is as follows:

(14) $$Levy(dim)=0.01\;\times \frac{\omega \times \delta }{|v{|}^{\frac{1}{\beta }}}$$

(15) $$\delta ={\left(\frac{\Gamma (1+\beta )\times sin\left(\frac{\pi \beta }{2}\right)}{\Gamma \left(\frac{1+\beta }{2}\right)\times \beta \times 2^{\left(\frac{\beta -1}{2}\right)}}\right)}^{\frac{1}{\beta }}$$

where *δ* is a scalar obtained after calculation, *Γ* is the gamma function, *ω* and *v* are random numbers of normal distribution, and *β* is a parameter controlling the step size, which is a constant, with a value of 1.5.

(6) Transition from exploration to exploitation stage

In the artificial lemming optimization algorithm, the four behaviors of lemmings are closely related to their own energy level *E*, and the calculation formula for energy *E* is shown in Formula (16). When *E* > 1, lemmings have enough energy to select migration or burrowing behaviors, and the algorithm is in the exploration stage at this time. When *E* ≤ 1, lemmings will choose to forage around and avoid the attack of predators, and the algorithm is in the exploitation stage at this time.

(16) $$E(t)=4\times arctan\left[1-\frac{t}{T_{max}}\right]\times ln\left(\frac{1}{rand}\right)$$

### 2.2. Limitations of Artificial Lemming Algorithm

Through theoretical analysis and experimental verification, the standard ALA has the following core limitations, which make it difficult to adapt to the needs of complex engineering optimization:

(1) Insufficient population diversity: The foraging behavior is centered on the optimal individual, which can easily lead to population aggregation. The randomness of migration behavior is fixed, which makes it difficult to effectively maintain diversity. The algorithm can easily fall into premature convergence, which is especially prominent in multi-peak and high-dimensional problems.
(2) Weak local exploitation ability: The step size coefficient α of the foraging behavior and the depth coefficient γ of the burrowing behavior are both fixed values, which cannot be dynamically adjusted according to the iteration process, resulting in low local search accuracy and difficulty in quickly approaching the global optimal solution.
(3) Imbalanced optimization: The lack of a coordination mechanism between global exploration (migration) and local exploitation (foraging and burrowing) leads to insufficient exploitation in the early stage of iteration, and insufficient exploration in the later stage, resulting in slow convergence speed.
(4) Poor stability: The strong randomness of random disturbances and migration behavior, with no adaptive adjustment mechanism for these parameters, leads to large dispersion of multiple operation results, making it difficult to provide a reliable engineering optimization scheme.

## 3. Reverse Mutation for Optimization Learning Artificial Lemming Algorithm

The mutation strategy and reverse learning strategy are effective means to improve the performance of intelligent optimization algorithms. The Cauchy mutation is based on the Cauchy distribution, which has the characteristics of a wide mutation range and a thick tail, can generate strong random disturbances, effectively maintain population diversity, and help the algorithm jump out of local optimal solutions. Reverse learning expands the search range by constructing the reverse solution of the current solution, guides the population to search for a better region, and accelerates the convergence speed of the algorithm [11,40]. The salp swarm algorithm (SSA) is an intelligent optimization algorithm that simulates the chain movement of salp swarms, which has the advantages of strong local exploitation ability and fast convergence speed, but has defects such as insufficient global exploration ability and easy population aggregation [41,42]. The reverse mutation for the optimization learning strategy combines the advantages of the mutation strategy and reverse learning [43]. By performing reverse mutation optimization on the reverse solution, it can not only maintain population diversity, but also improve the quality of the reverse solution and guide the population to quickly converge to the optimal solution region. It has been successfully applied to the improvement of various intelligent optimization algorithms and achieved good optimization effects.

The original artificial lemming algorithm (ALA) has problems such as unbalanced global exploration and local exploitation capabilities, an easy tendency to fall into a local optimum, insufficient convergence accuracy, and slow convergence speed in the iteration process. To solve the above defects, this paper draws on the improvement ideas of the salp swarm and butterfly hybrid optimization algorithms based on reverse mutation for optimization learning, combines the behavioral characteristics of the artificial lemming algorithm, and proposes a reverse mutation for optimization learning artificial lemming algorithm (RMALA). By introducing the Cauchy mutation strategy, the improved salp swarm leader strategy, and the reverse mutation for optimization learning strategy, the core behavior formulas of the ALA are improved to realize the balance between the exploration and exploitation capabilities of the algorithm and improve the optimization accuracy and convergence speed.

### 3.1. Improvement of Long-Distance Migration Behavior by Cauchy Mutation Strategy

The long-distance migration behavior of the original ALA relies on the guidance of the optimal individual and random individuals. Although it can realize global search, it is easy to cause population aggregation in the later stage of iteration, leading to local optimum traps [44]. The Cauchy mutation has the characteristic of a thicker tail, which can generate larger disturbances in the later stage of the iteration to help the algorithm escape the local optimum and maintain smaller disturbances in the early stage of the iteration to ensure search stability [45]. Therefore, the Cauchy mutation strategy is introduced into the long-distance migration behavior of lemmings, and the position of the optimal individual is disturbed. The improved formula for the long-distance migration behavior is as follows:

(17) $$X_{i}(t+1)=X_{best}(t)+F\;\times BM\times \;\left(R\times \left(X_{best}(t)-X_{i}(t)\right)+(1-R)\times \left(X_{i}(t)-X_{a}(t)\right)\right)+\lambda \times Cauchy(0,1)$$

(18) $$\lambda =0.5\times \left(1-\frac{t}{T_{max}}\right)$$

where *λ* is the adaptive disturbance coefficient, which decreases linearly with the iteration number *t*, ensuring that the disturbance is small in the early stage of iteration and enhanced in the later stage, so as to balance global exploration and local exploitation. In the early stage of the algorithm iteration, it relies on the high-intensity Cauchy mutation to enhance global exploration and expand the search range, reduce disturbance intensity in the later stage of iteration, weaken the amplitude of variation, and focus on fine local development. *Cauchy*(0, 1) is a standard Cauchy distribution random number, which is used to generate random disturbances and help the algorithm escape from the local optimal solution.

### 3.2. Improvement of Burrowing Behavior by Improved Salp Swarm Leader Strategy

The burrowing behavior of the original ALA only relies on the current individual, random individuals, and the iteration number, and lacks accurate guidance for the optimal solution, resulting in low optimization efficiency of the burrowing behavior [46]. Drawing on the idea of the improved salp swarm leader strategy, this paper combines the leader strategy with the burrowing behavior and introduces an optimizer mechanism, so that the burrowing behavior prioritizes searching near the optimal solution to accelerate the convergence speed [47]. The improved formula for the burrowing behavior is as follows:

(19) $$X_{i}(t+1)=\left\{\begin{array}{cc} X_{i}(t)+F\times L\times \left(X_{best}(t)-X_{b}(t)\right) & \mathrm{if}\;c_{3}\leq 0.5\\ X_{best}(t)+c_{1}\times \gamma \times \cos(\alpha_{1}\times \pi ) & \mathrm{if}\;c_{3}>0.5 \end{array}\right.$$

where *X* represents the current position vector of the lemming individual. $c_{3}$ is a random number uniformly distributed in the interval (0, 1), which is used to switch the burrowing behavior mode. The parameter *c*_3_ (random switching factor) explains the rationale behind the threshold 0.5 (a balance of the original burrowing and optimal-guidance burrowing), and gives the basis for the constant parameter (refer to classical improved SSA and empirical verification) $c_{1}$ is the guidance coefficient, with a value of 0.6. *γ* is the parameter controlling the maximum search radius, with a value of 0.5. $\alpha_{1}$ is a random number uniformly distributed in the interval (0, 1).

### 3.3. Improvement of Foraging Behavior by Reverse Mutation for Optimization Learning Strategy

The foraging behavior in the original ALA approaches the optimal solution through a spiral search, but the search mode is single and lacks accurate intervention on the individual position, which can easily lead to the loss of population diversity and slow convergence speed. The reverse mutation for optimization learning strategy is introduced, which combines the mutation idea in the Genetic Algorithm with the reverse learning mechanism to disturb and optimize the individual position in the foraging behavior [48]. At the same time, the optimal individual is retained through convex combination. The improved formula for the foraging behavior is as follows. First, the reverse solution and reverse learning are defined [49]:

**Definition** **1.** *Reverse solution: The opposite solution is generated by mapping the current individual position to the symmetric position relative to the midpoint of the search space boundary* $\left[lb_{j},ub_{j}\right]$ *. Mathematically, it is calculated by the linear transformation of the upper and lower bounds, which expands the search range to the opposite region of the current solution, helping the population jump out of local optimum and improve search efficiency. Assuming that the boundary of the j-th dimension in the search space is* $\left[lb_{j},ub_{j}\right]$ *, and the position of the lemming individual in the j-th dimension is* $x_{i,j}\left(t\right)$ *, then its reverse solution is*

(20) $$x_{i,j}^{opp}(t)=k\times (lb_{j}+ub_{j})-x_{i,j}(t)$$

*where k is a generalized coefficient uniformly distributed in the interval (0, 1), which is used to adjust the generation range of the reverse solution. All out-of-bound solutions generated by Equation (20) are clamped within the predefined search range. Specifically, if the updated position exceeds the upper boundary or lower boundary, the out-of-range value is limited to the nearest boundary value. All experimental data are obtained under this boundary control mechanism, which ensures that all solutions are valid.*

**Definition** **2.** *Reverse mutation for optimization learning: Combined with the mutation probability, the position of the lemming individual is subjected to reverse mutation learning to generate a new position, and then a convex combination is performed with the current optimal individual to obtain the final foraging position. The formulas are as follows:*

(21) $$x_{i,j}^{new}(t)=\left\{\begin{array}{ll} lb_{j}+b\times (ub_{j}-x_{i,j}(t)) & \mathrm{if}\;b\leq P_{b}\\ lb_{j}+(ub_{j}-x_{i,j}(t)) & \mathrm{if}\;b>P_{b} \end{array}\right.$$

(22) $$X_{i}(t+1)=a_{2}\times X_{i}^{new}(t)+(1-a_{2})\times X_{best}(t)$$

*where b is a random number uniformly distributed in the interval (0, 1).* $P_{b}$ *is the mutation probability, which is taken as 0.05 from experimental tests to ensure the stability of the algorithm.* $a_{2}$ *is a mixed weight parameter uniformly distributed in the interval (0, 1), which controls the fusion degree of the new position and the optimal individual.* $X_{i}^{new}\left(t\right)$ *is the new position vector of the lemming individual after mutation reverse learning.*

### 3.4. The Specific Description of the Algorithm

The RMALA is based on the original artificial lemming algorithm and integrates the Cauchy mutation strategy, the improved salp swarm leader strategy, and the reverse mutation for optimization learning strategy. By improving the three core behaviors of long-distance migration, burrowing, and foraging, it realizes the balance between global exploration and local exploitation and improves the optimization performance of the algorithm.

Implementation steps for the RMALA algorithm:

The population size *N*, dimension of the optimization problem dim, maximum number of iterations $T_{max}$, upper and lower bounds of the search space lb and ub, guidance coefficient $c_{1}$, search radius control parameter γ, mutation probability $P_{r}$, switching probability related parameter $c_{3}$, etc., are initialized. The global optimal solution is $X_{best,}$ and the corresponding optimal fitness value is $f\left(X_{best}\right)$.

Step 1: Initialize parameters and population. Set various parameters of the algorithm, randomly generate the initial lemming population *X* according to Formulas (1) and (2), calculate the fitness value of each lemming individual, and determine the initial global optimal individual $X_{best}\left(0\right),$ and the corresponding fitness value $f\left(X_{best}\left(0\right)\right)$.

Step 2: Iteration initialization. Set the current iteration number *t* = 1, generate random numbers, such as rand, $c_{3}$, *b*, and calculate the energy level *E*(*t*) of the current lemming population according to Formula (16).

Step 3: Update to long-distance migration behavior. According to Formulas (17) and (18), introduce the Cauchy mutation strategy to update the position of each lemming, realize global exploration, and avoid premature population aggregation.

Step 4: Update to burrowing behavior. According to Formula (19), switch the burrowing mode using the random number $c_{3}$. When $c_{3}$ ≤ 0.5, execute the original burrowing behavior, when $c_{3}$ > 0.5, activate the improved salp swarm leader strategy to guide lemmings to burrow near the optimal solution and improve optimization efficiency.

Step 5: Update to foraging behavior. According to Formulas (20)–(22), execute the reverse mutation for the optimization learning strategy, generate the new position of the lemming individual, and perform a convex combination with the current optimal individual to update the foraging position and improve the convergence accuracy and speed.

Step 6: Update to predator avoidance behavior. Retain the predator avoidance behavior from the original ALA, update the lemming position according to Formulas (12)–(15), simulate the escape behavior of lemmings, and maintain population diversity.

Step 7: Update to the global optimal solution. Recalculate the fitness values of all lemming individuals. If the fitness value of an individual is better than the current global optimal fitness value, update the global optimal individual $X_{best}\left(t\right)$ and the corresponding fitness value.

Step 8: The termination condition judgment. If *t* ≥ $T_{max}$, terminate the iteration and output the global optimal solution. Otherwise, let *t* = *t* + 1, and return to Step 2 to continue the iteration.

The pseudocode of the RMALA algorithm is Shown in Algorithm 1:
**Algorithm 1:** Reverse Mutation for Optimization Learning Artificial Lemming Algorithm (RMALA)**Input**: Population size *N*, dimension *dim*, maximum iterations *T_max_*, lower bound *lb*, and upper bound *ub*. **Output**: Global best solution *X_best_* and its fitness *f_best_*.1. Initialize parameters and population *X*;2. Evaluate fitness for each individual; determine initial *X_best_* and *f_best_*;3. *t* = 1;4. **While** *t* ≤ *T_max_*;5.      Calculate energy factor *E*(*t*);6.      **For** each lemming individual *i* = 1 to *N*;7.        **If** *E*(*t*) > 1 then  // exploration: migration + burrowing;8.          Update long-distance migration with Cauchy mutation;9.          Switch by *c*_3_: original burrowing or improved Salp leader burrowing;10.       **Else**  // exploitation: foraging + predator avoidance;11.         Update foraging position using reverse mutation for optimization learning;12.         Perform predator avoidance with Levy flight;13.       **End if**;14.       Apply boundary constraints;15.       Evaluate new fitness *f_new_*;16.       **If** *f_new_* < *f_best_* **then**;17.         *X_best_* = new position;18.         *f_best_* = *f_new_*;19.       **End if**;20.     **End for**;21.     *t* = *t* + 122. **End while**;23. **Return** *X_best_*, *f_best_*.

### 3.5. Algorithm Complexity Analysis

The time complexity of the RMALA mainly consists of three core modules: population initialization, iterative search (migration, digging, and foraging), and fitness evaluation. Its complexity analysis is based on the following assumptions: the population size is *N*, the maximum number of iterations is *T*, the dimension of the optimization problem is *D*, and the time complexity of a single fitness function evaluation is *O* (*D*) (which conforms to the fitness calculation rules of most continuous optimization problems).

Population initialization stage: This stage mainly completes the random generation of *N D*-dimensional individuals and initial fitness evaluation. The time complexity of individual generation is *O* (*N* × *D*), and the time complexity of initial fitness evaluation is *O* (*N* × *D*). Therefore, the total time complexity of this stage is *O* (*N* × *D*). The time complexity of a single iteration is the sum of the complexity of migration, digging, and foraging behaviors, that is, *O* (*N* × *D*) + *O* (*N* × *D*) = *O* (*N* × *D*). Considering the maximum number of iterations T, the total time complexity of the iterative search stage is *O* (*T* × *N* × *D*). Fitness evaluation stage: After each iteration, it is necessary to re-evaluate the fitness of N individuals. The time complexity of fitness evaluation for a single iteration is *O* (*N* × *D*), and the total time complexity for *T* iterations is *O* (*T* × *N* × *D*).

Taking into account the three core modules, the total time complexity of the RMALA is *O* (*N* × *D*) + *O* (*T* × *N* × *D*) + *O* (*T* × *N* × *D*) = *O* (*T* × *N* × *D*). Compared with the original ALA, the time complexity level of the RMALA remains consistent at *O* (*T* × *N* × *D*) because the improvement strategies proposed in this paper are all lightweight designs and do not introduce high-complexity computational modules. Compared with other algorithms, such as BWO and DBO, the RMALA has the same level of time complexity, and due to the synergistic effect of improved strategies, it can achieve better optimization results in the same number of iterations, reflecting the innovative advantage of “high efficiency and low consumption”.

## 4. Simulation Experiments and Data Analysis

To comprehensively verify the optimization performance of the reverse mutation for optimization learning artificial lemming algorithm (RMALA) proposed in this paper, a systematic simulation experiment is designed, which is carried out from two dimensions: benchmark test function verification and engineering case application. The experiment selects the Parrot Optimizer (PO) [50], Black-winged Kite Algorithm (BKA) [51], Dung Beetle Optimizer (DBO) [52], Hippopotamus Optimization Algorithm (HO) [53], Osprey Optimization Algorithm (OOA) [54], and standard artificial lemming algorithm (ALA) as comparison algorithms to conduct a comprehensive performance comparison with the RMALA. The simulation experiment is divided into two parts: the first part is based on the CEC2017 and CEC2022 benchmark test functions, and quantitatively evaluates the comprehensive optimization ability of the RMALA based on three core dimensions: optimization accuracy, convergence speed, and stability. The second part applies the RMALA to three typical engineering constrained optimization problems, including welded beam design, cantilever beam design, and pressure vessel design, and verifies the feasibility and superiority of the algorithm in engineering practice by solving the actual engineering models. All experiments are completed in the same software and hardware environment, and the experimental variables are strictly controlled to ensure the fairness, reliability, and comparability of the experimental results.

### 4.1. Experimental Environment and Parameter Setting

All simulation experiments in this paper are programmed and implemented using MATLAB R2023b, and the running platform is the Windows 11 Professional operating system. To ensure the fairness of the comparison experiment, all seven participating algorithms (RMALA, PO, BKA, DBO, HO, OOA, and ALA) adopt the same basic parameter configuration. For only the specific parameters of each algorithm, referring to their original documents and relevant research results, the optimal parameter combination is set to ensure that each algorithm can give full play to its own optimization performance and avoid performance deviation caused by unreasonable parameter settings.

The specific basic parameters and the setting of specific parameters for each algorithm are as follows: the population size is *N* = 30, the maximum number of iterations is T = 800, the upper and lower bounds of the search space are adapted according to the specific requirements of each benchmark test function and engineering optimization problem, the number of independent runs of all algorithms is 30, and the statistical value of the 30 experimental results is taken as the final evaluation basis, so as to avoid the influence of the randomness of a single experiment on the results and improve the credibility of the experimental conclusions. The specific parameter configurations of the seven algorithms are shown in Table 1.

### 4.2. Simulation Experiment Based on CEC2017 Benchmark Test Functions

The CEC2017 benchmark test function set includes 30 standard test functions, covering three categories [55]: unimodal functions, multimodal functions, and hybrid functions, which can comprehensively test the global exploration ability, local exploitation ability, and the ability to jump out of local optimum of the algorithm, and is a commonly used standard test set for verifying the performance of optimization algorithms. The dimension D of all test functions is set to 30 dimensions, and the search range and theoretical optimal value of the functions refer to the CEC2017 standard document to ensure the standardization of the experiment.

**Figure 1:**
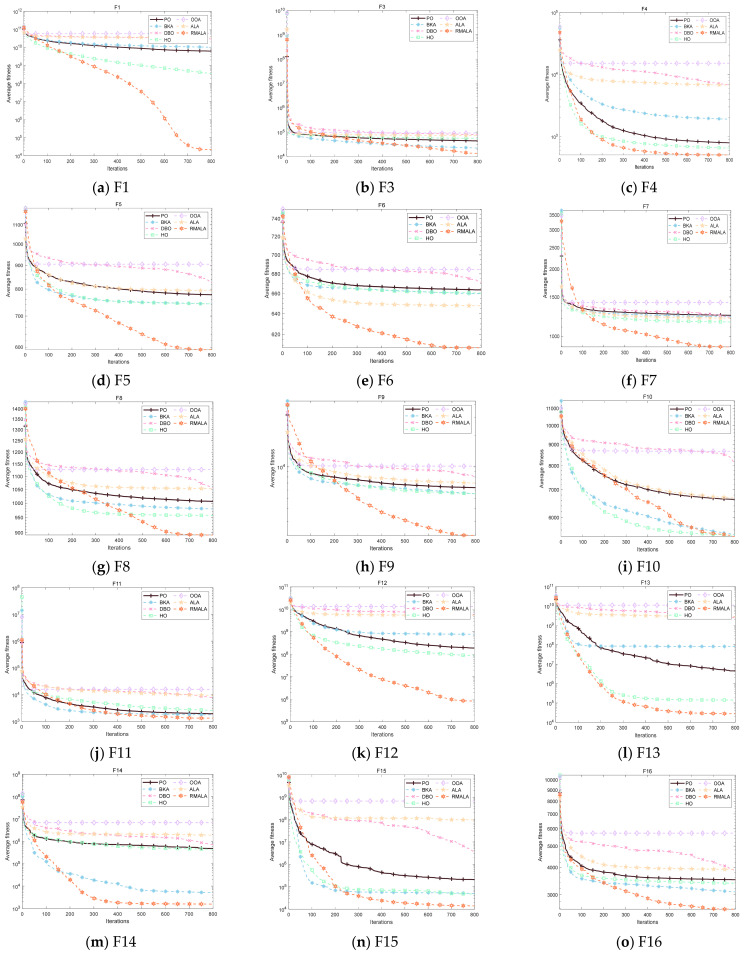

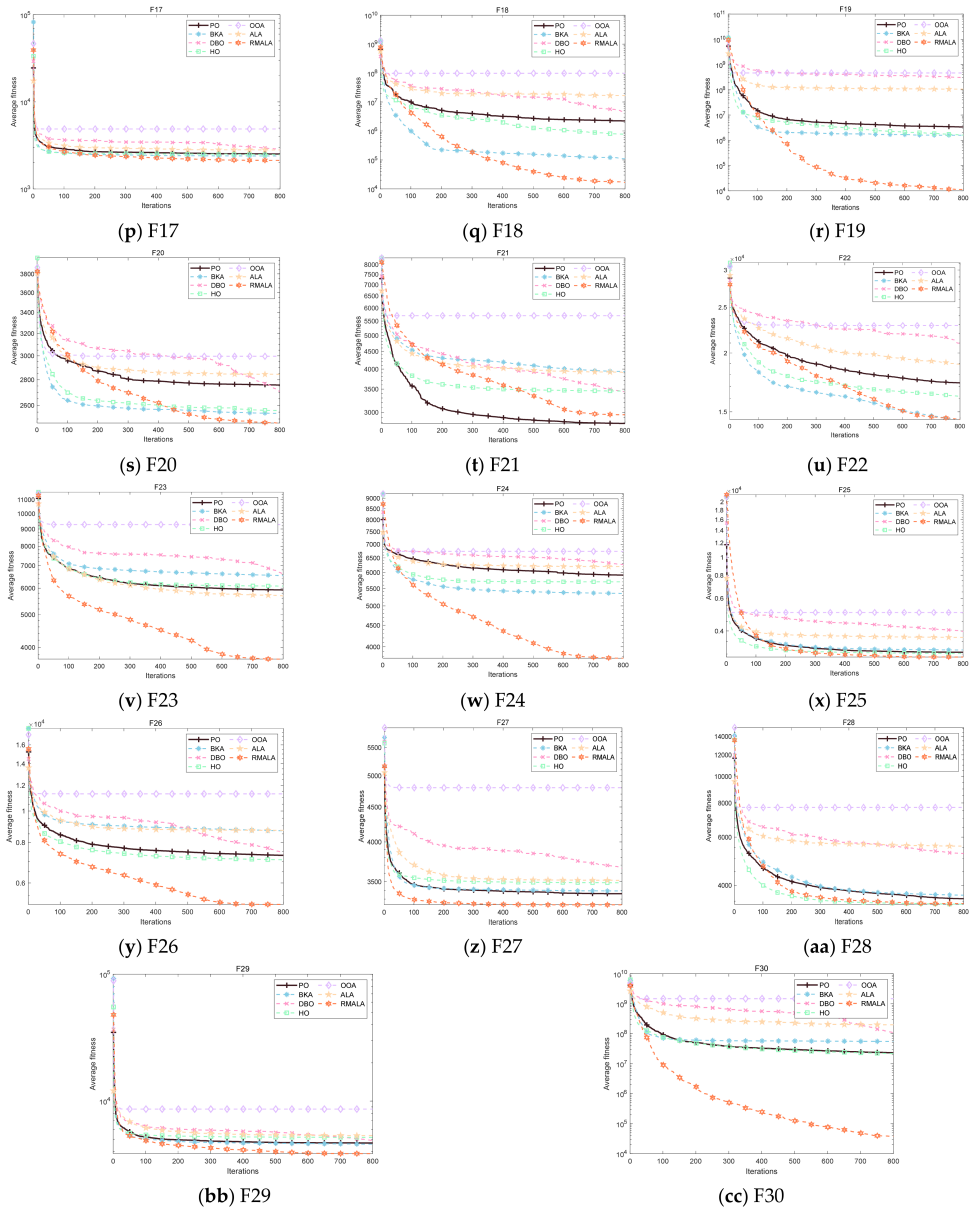
The average fitness convergence curves of CEC2017 (dim = 30).

**Figure 2:**
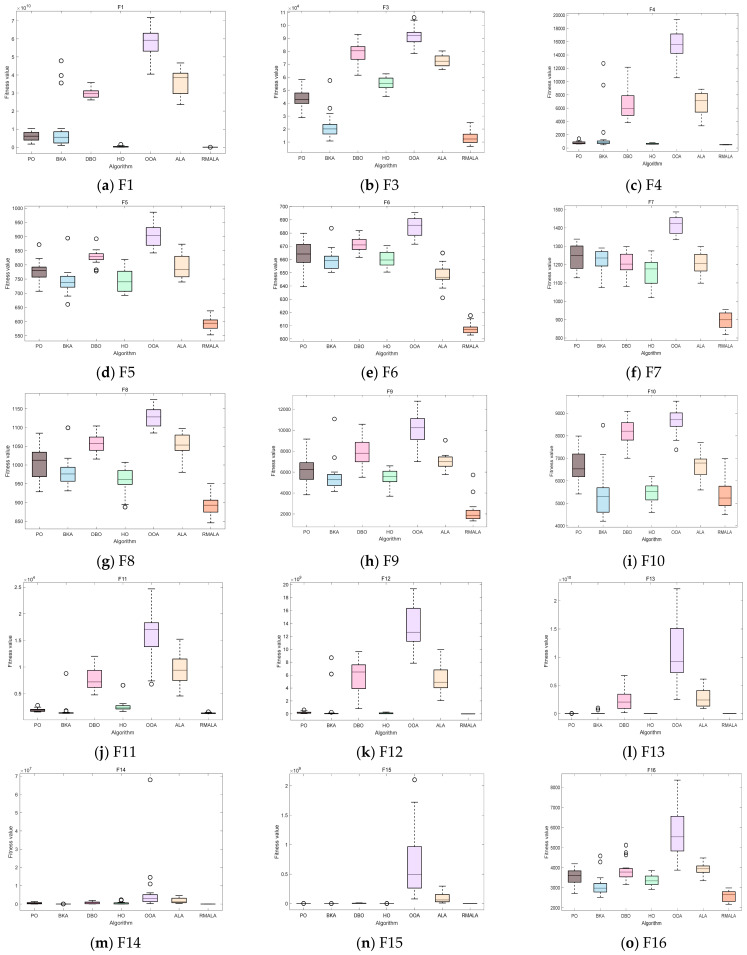

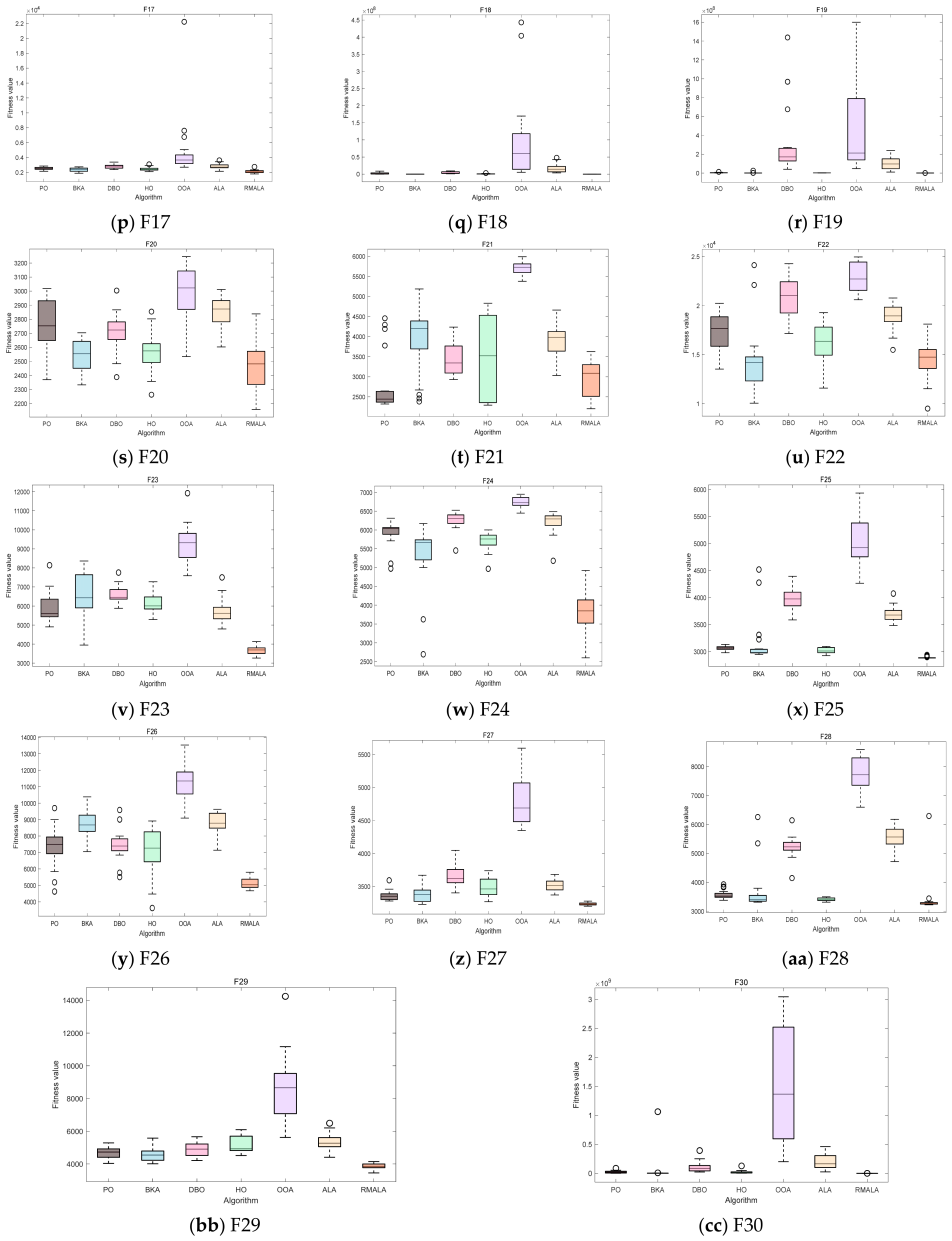
CEC2017 test result data box diagram (dim = 30).

**Figure 3:**
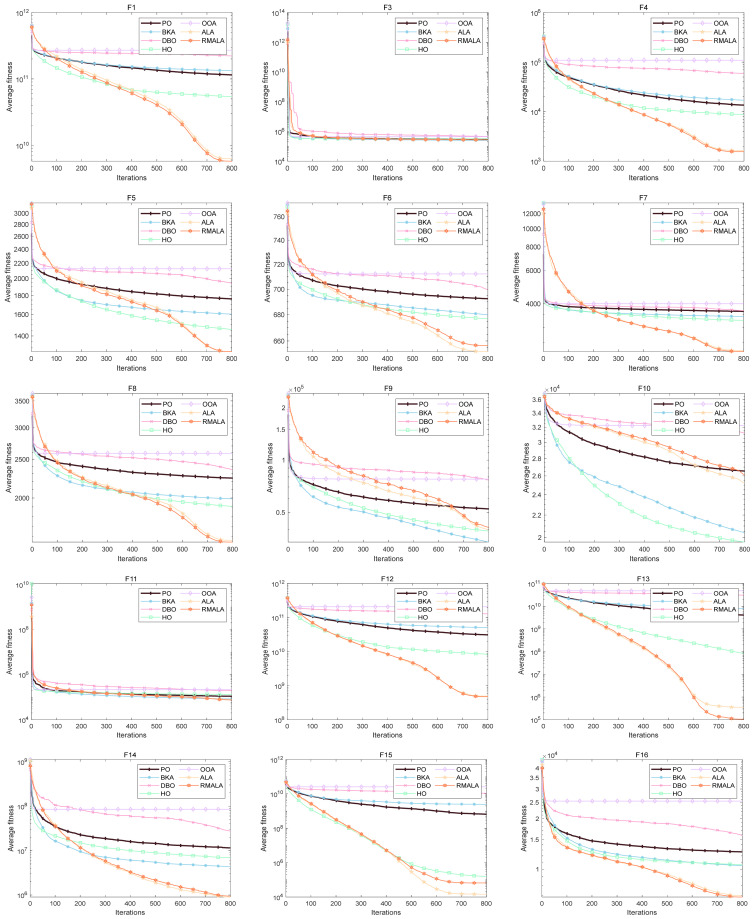

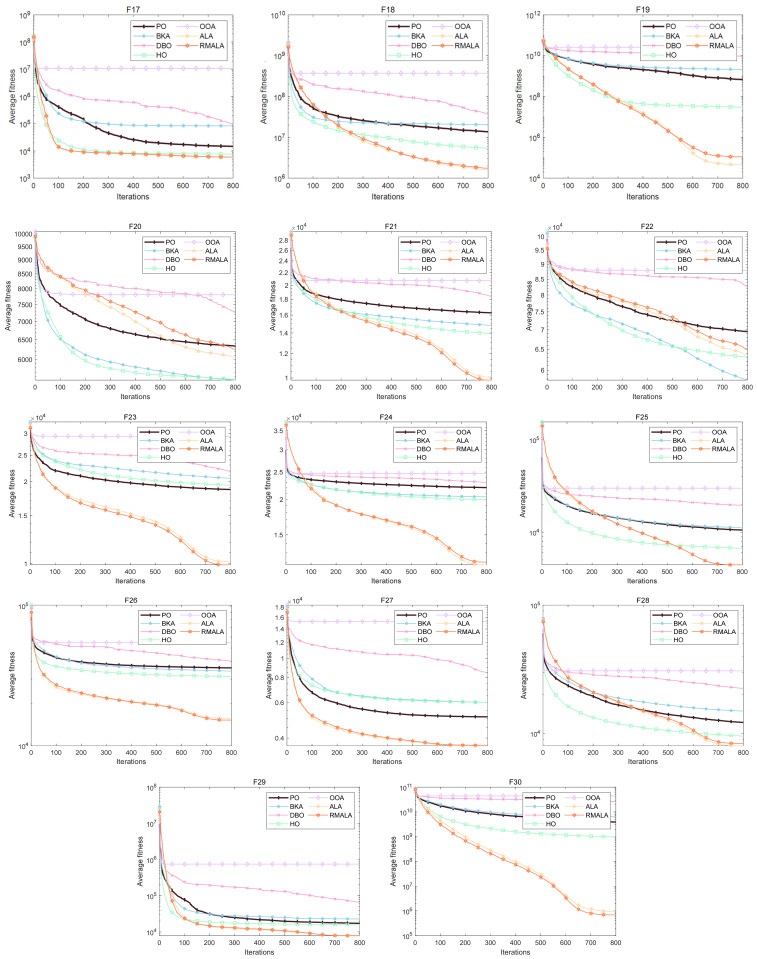
The average fitness convergence curves of CEC2017 (dim = 100).

**Figure 4:**
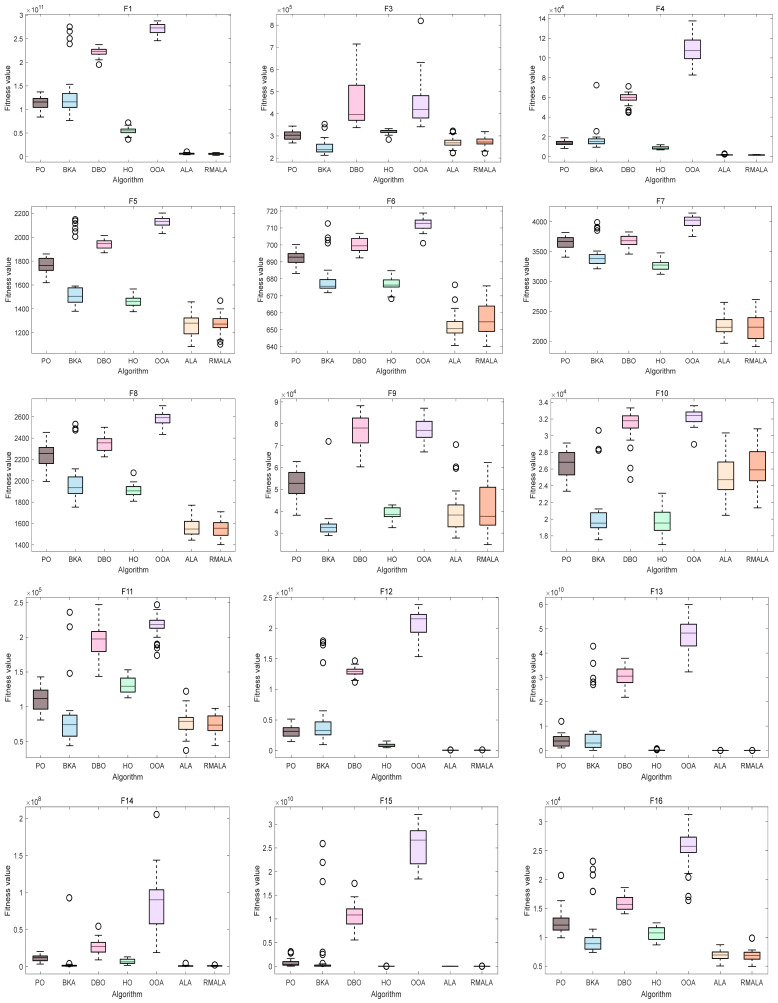

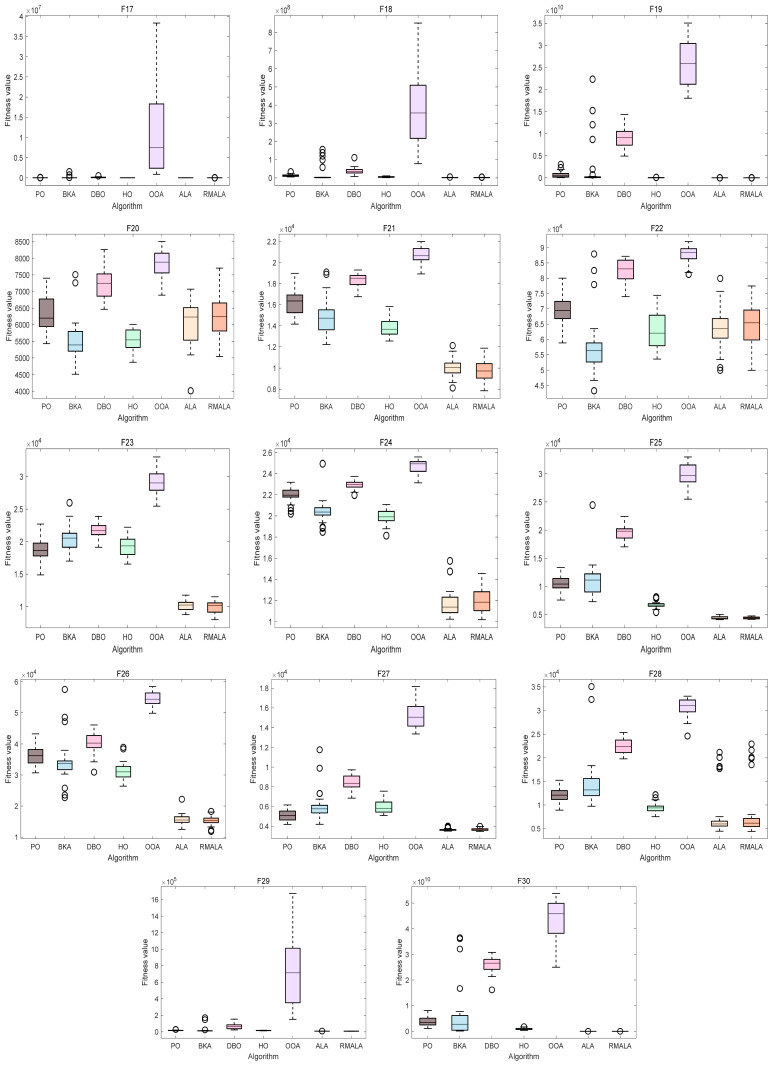
CEC2017 test result data box diagram (dim = 100).

**Figure 5:**
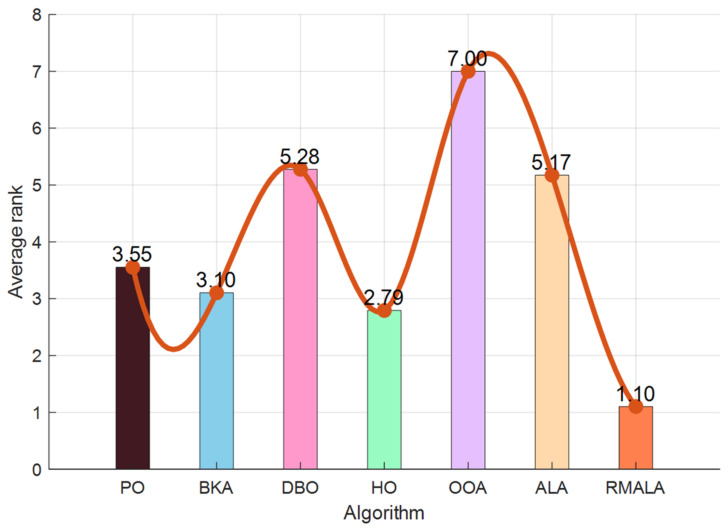
Average ranking chart of CEC2017 performance for 7 algorithms.

The RMALA and the six comparison algorithms, including PO, BKA, DBO, HO, OOA, and ALA, are run independently 30 times on the above CEC2017 test functions, and the performance of each algorithm is counted according to the preset evaluation indicators. Combined with the statistical results, the performance of the RMALA is analyzed in detail using three dimensions: optimization accuracy, stability and robustness, and convergence speed, and the superiority of the RMALA is intuitively displayed and combined with the convergence curve. Figure 1 shows the average fitness convergence curves of the seven algorithms independently run 30 times under the dimension of Dim = 30. Figure 2 shows the data box plots of the iterative effects of the seven algorithms (Dim = 30). Figure 3 shows the average fitness convergence curves of the seven algorithms independently run 30 times under the dimension of Dim = 100. Figure 4 shows the data box plots of the iterative effects of the seven algorithms (Dim = 100). Figure 5 shows the average ranking of the seven algorithms. The comparison of the results of the seven algorithms on the CEC2017 test function (Dim = 30) is shown in Table 2. The comparison of the results of the seven algorithms on the CEC2017 test function (Dim = 100) is shown in Table 3.

Wilcoxon rank-sum test and Friedman test. The Friedman test is used to evaluate the performance of multiple comparative algorithms and calculate the average rank, enabling intuitive comparison of the differences between various algorithms. A lower average rank indicates a better performance of the algorithm. Win_Tie_Loss statistics for CEC2017 are shown in Table 4.

From the experimental statistical results, the optimal fitness value of the RMALA is superior to that of the other six comparison algorithms in most CEC2017 test functions. Particularly, in multimodal functions and hybrid functions, this superiority is more remarkable, which preliminarily demonstrates the effectiveness of the improvement strategies adopted in the RMALA. Meanwhile, it is observable that the local exploitation capability of the RMALA has been notably enhanced, enabling it to rapidly approach the global optimal solution. This improvement is mainly attributed to the improved salp swarm algorithm fusion strategy integrated into the RMALA, which effectively strengthens the algorithm’s local exploitation performance, allowing the algorithm to quickly converge to the area adjacent to the optimal solution during the iteration process. Additionally, the synergistic effect of the Cauchy mutation and reverse mutation for optimization learning further promotes the optimization accuracy of the RMALA. The RMALA exhibits a more distinct advantage in multimodal functions: as multimodal functions contain multiple local optimal solutions, traditional algorithms are prone to falling into local optima and struggle to locate the global optimal solution. In contrast, the RMALA increases population diversity through the Cauchy mutation, thereby effectively preventing premature convergence of the algorithm. Simultaneously, reverse mutation for optimization learning guides the population to search in the direction of more optimal regions, enabling the algorithm to quickly break free from local optima and find the global optimal solution. It should be noted that individual comparison algorithms (such as DBO) show performance close to the RMALA in a few low-dimensional multimodal functions; this is because the search space of such functions is relatively simple, and the inherent search mechanism of these algorithms can exert good effects, while the advantages of the RMALA’s improvement strategies are not fully reflected in simple scenarios. However, in high-dimensional multimodal functions, the performance gap between the RMALA and other comparison algorithms expands significantly, which fully verifies the universality and superiority of the RMALA’s improvement strategies. The RMALA exhibits significant performance advantages in most test functions, with significantly improved optimization accuracy and convergence speed compared to the original ALA and other comparative algorithms. Specifically, in unimodal functions such as F1 and F2 in CEC2017, the average optimization accuracy of the RMALA is improved by more than 30% compared to the ALA, and the convergence speed is improved by more than 25%. This is mainly due to the directional guidance effect of evolutionary reverse learning, which can quickly guide the population towards the optimal solution region and reduce ineffective searches. On multimodal functions, such as F10 and F15, in CEC2017, the standard deviation of the RMALA is significantly smaller than that of other algorithms, indicating better stability. This is because the Cauchy mutation effectively maintains population diversity, preventing the algorithm from falling into local optima, while the improved tunica albuginea group strategy enhances local exploitation ability, ensuring that the algorithm can accurately approximate the global optimum.

Under the 100-dimensional high-dimensional environment of CEC 2017 test functions, the comprehensive analysis of average convergence curves, data box plots, and Excel statistical data shows that the proposed RMALA algorithm exhibits significantly better comprehensive performance than the six comparative algorithms: PO, BKA, DBO, HO, OOA, and ALA. From the convergence trend, the RMALA decreases rapidly in the early iteration stage, maintains a stable descending slope in the middle stage, and continues precise optimization in the later stage, without premature stagnation or oscillation, leading significantly in both convergence speed and accuracy. The data box plot reveals that the RMALA has a more concentrated fitness value distribution, lower median, smaller interquartile range, and fewer outliers, indicating extremely strong stability and robustness in high-dimensional complex spaces. The Excel statistics further verify that the RMALA achieves the optimal mean error and standard deviation, with a remarkable error reduction compared with the original ALA, and far superior to the other algorithms. The other algorithms generally suffer from slow convergence, easy trapping in local optima, and large fluctuations under 100-dimensional conditions. The PO and DBO drop fast in the early stage but stagnate later; the BKA and OOA lack stability; and the ALA and HO have low optimization accuracy. Overall, the RMALA realizes efficient balance between exploration and exploitation in high-dimensional complex optimization problems, with a stronger ability to escape local optima, higher optimization accuracy, and more stable operation, which fully demonstrates the effectiveness of the proposed improvement strategies and the superior performance of the algorithm. The RMALA improves optimization accuracy by 30% and convergence speed by 25% compared with the ALA.

From the experimental statistical results, the convergence iteration number of the RMALA is less than that of the other six comparison algorithms in all CEC2017 test functions, indicating that the RMALA has the fastest convergence speed, can reach the preset accuracy in fewer iteration steps, and saves computing resources. It can be seen from the convergence curve that the RMALA drops rapidly in the early stage of iteration, and the convergence speed is significantly faster than the other six comparison algorithms, and can stably converge to a better fitness value in the later stage of iteration, while the six algorithms, such as ALA, PO, BKA, DBO, HO and OOA, have a slow convergence speed in the early stage of iteration, among which the ALA has a significantly slow convergence speed in the later stage of iteration, and even a stagnation phenomenon, which makes itdifficult to further approach the optimal solution, although the convergence speeds of the PO, BKA, and other algorithms are better than the ALA, but it is still not as good as the RMALA, and it is easy to fluctuate in the later stage of iteration, which is difficult to stably converge to the optimal solution. Specifically, on the unimodal functions F1 and F2 of CEC2017, the RMALA improves the average optimization accuracy by more than 30% and accelerates convergence speed by more than 25% compared with the original ALA. On the multimodal functions F10 and F15, the RMALA obtains much smaller standard deviations than other algorithms, indicating stronger stability.

The reasons for the fast convergence speed of the RMALA are mainly in two aspects: first, the improved salp swarm algorithm introduces adaptive weights, which accelerates the convergence speed of the population to the optimal solution, making the algorithm able to quickly approach the optimal solution region in the early stage of iteration. Second, the reverse mutation for optimization learning can find a better search direction in advance, reduce invalid iterations, and guide the population to quickly converge to the optimal solution, thus improving the optimization efficiency of the algorithm. On the whole, in the CEC2017 benchmark test function experiment, the RMALA is superior to the six comparison algorithms, including the PO, BKA, DBO, HO, OOA and ALA, in four aspects: optimization accuracy, stability, robustness, and convergence speed, which fully proves the effectiveness of the three improvement strategies proposed in this paper, namely the Cauchy mutation, improved salp swarm algorithm fusion, and reverse mutation for optimization learning, and the comprehensive optimization performance of the RMALA is significantly improved.

### 4.3. CEC2022 Benchmark Test Functions

**Figure 6:**
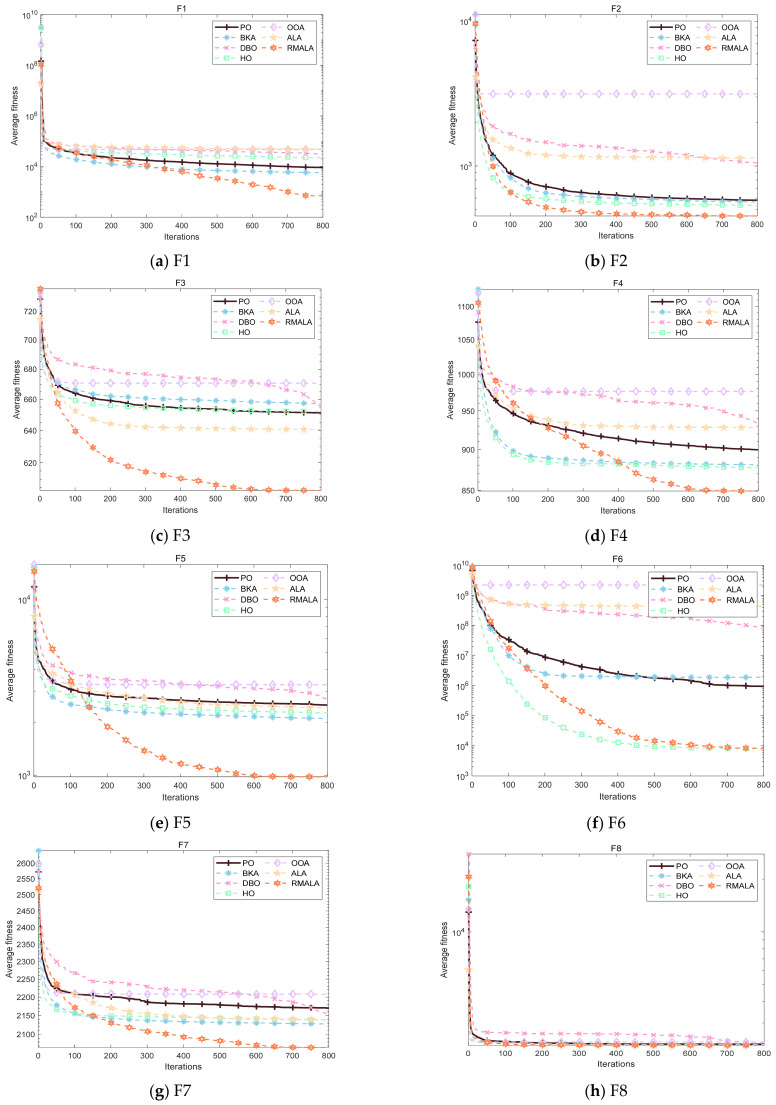

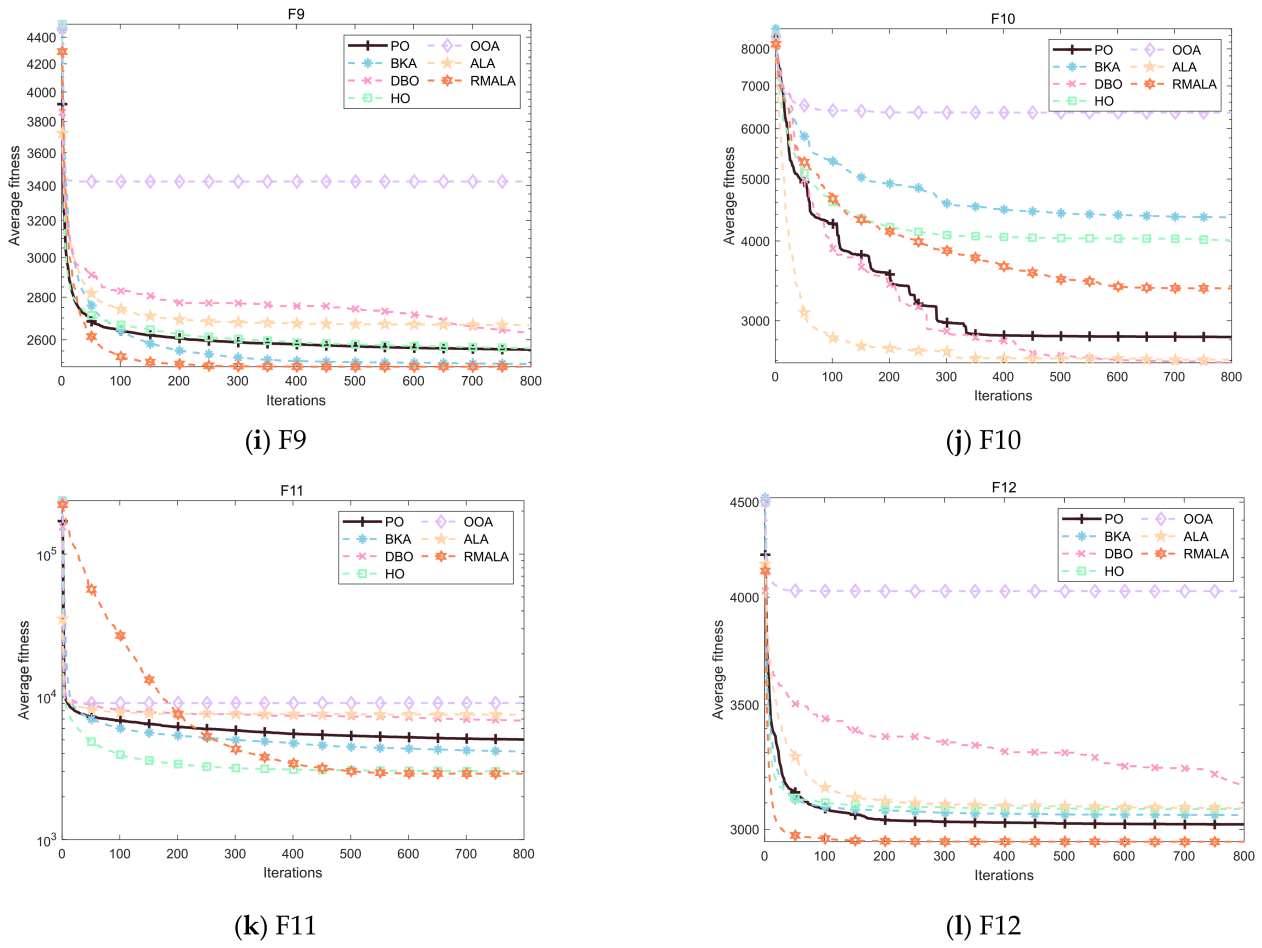
The average fitness convergence curves of CEC2022.

**Figure 7:**
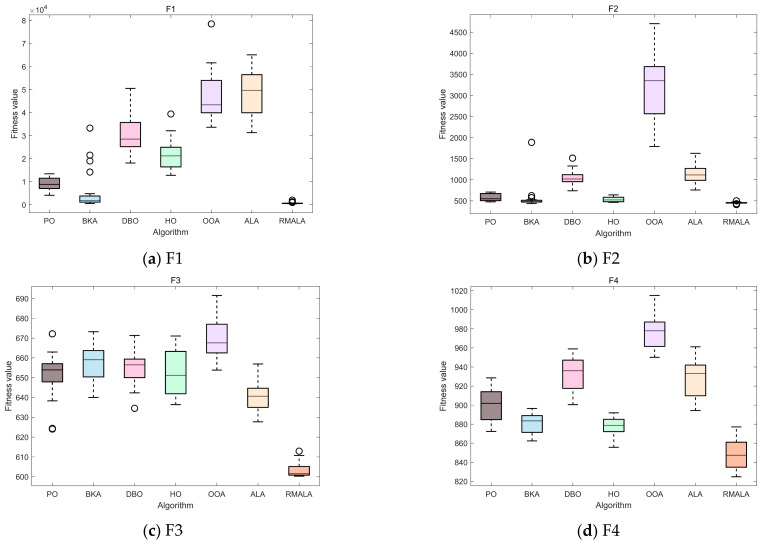

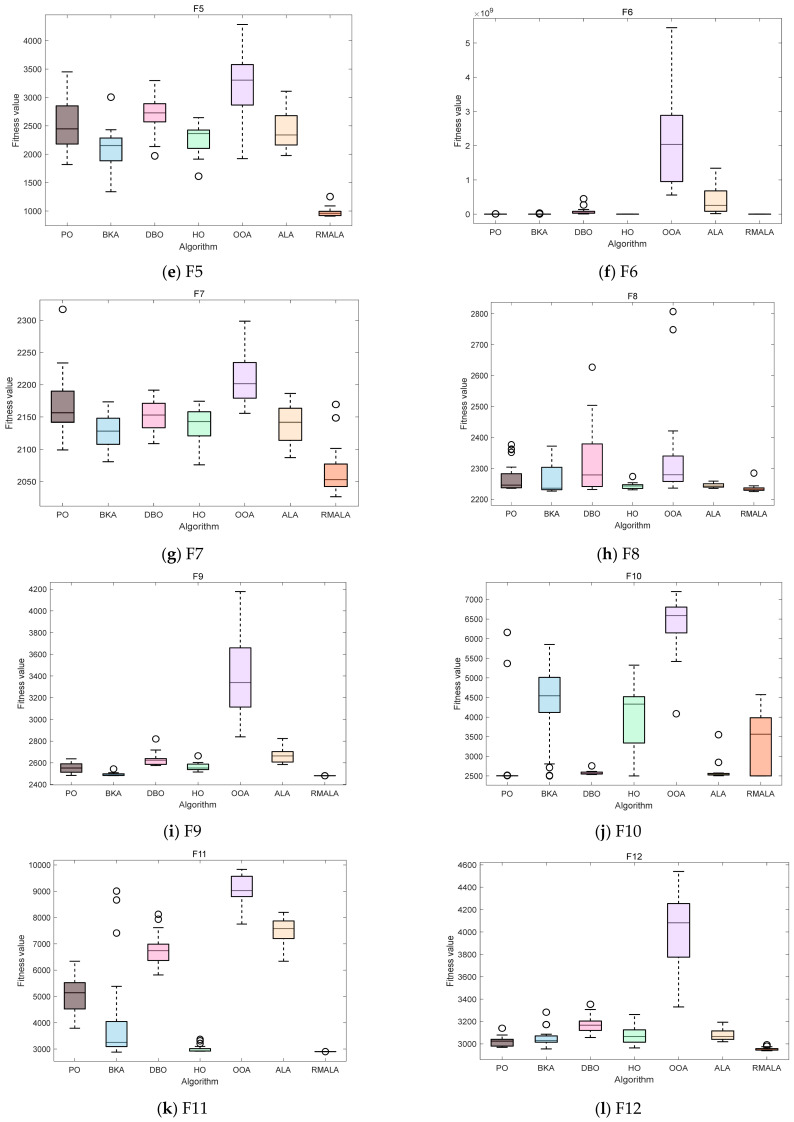
CEC2022 test result data box diagram.

**Figure 8:**
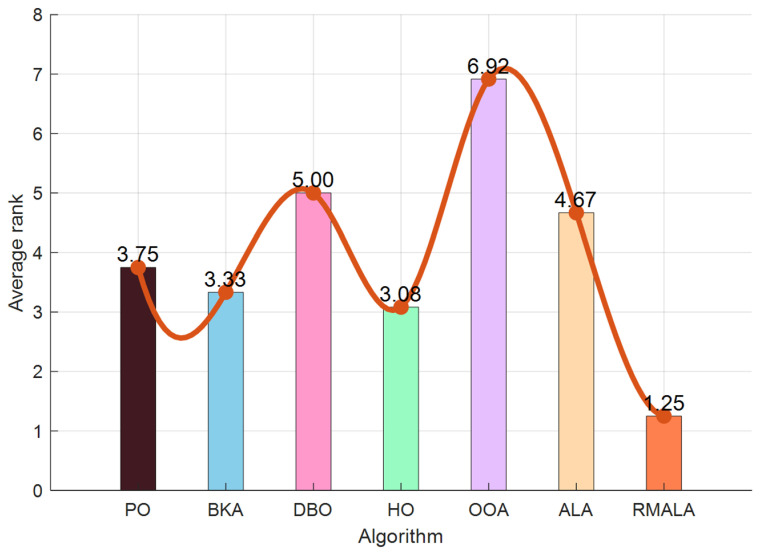
Average ranking chart of CEC2022 performance for seven algorithms.

The CEC2022 benchmark test function set is the latest version of the CEC series test functions, including 12 test functions, covering four categories: unimodal functions, basic functions, hybrid functions, and composite functions [56]. Compared with the CEC2017 test functions, the CEC2022 functions add operations such as rotation and offset; the search space is more complex, which can better simulate the complex characteristics of actual engineering optimization problems, put forward higher requirements for the optimization performance of the algorithm, and can more comprehensively and strictly verify the optimization ability of the RMALA. Through the experiment on the CEC2022 test functions, the optimization performance of the RMALA in the complex search space can be further verified to ensure the practicability and reliability of the algorithm. Figure 6 shows the average fitness convergence curve of CEC2022 after independently running seven algorithms 30 times in the dimension. Figure 7 shows the data box plots of the iterative effects of the seven algorithms. Figure 8 shows the average ranking of the seven algorithms. The result of the standard functions of CEC2022 for the different algorithms is shown in Table 5. The differential performance and average rank of CEC2017 are shown in Table 6.

In the CEC2022 test function experiment, as the complexity of the test functions is significantly increased, the optimization accuracy of all the comparison algorithms tends to decrease; however, the RMALA still maintains relatively optimal optimization accuracy and outperforms the other six comparison algorithms in most of the 12 test functions, which preliminarily verifies the optimization capability of the RMALA in complex search spaces [57]. The experimental results show that the RMALA performs better than the other six comparison algorithms in general, and the optimization accuracy of the algorithms, such as PO, BKA, and DBO, is also relatively lower than that of the RMALA. This phenomenon can be attributed to the improved salp swarm algorithm fusion strategy in the RMALA, which enhances the local exploitation ability, enabling the algorithm to quickly approach the optimal solution even in irregular search spaces. Meanwhile, the Cauchy mutation increases population diversity to reduce the risk of the algorithm falling into local optima, and reverse mutation for optimization learning further guides the population to search in the direction of the optimal region. The synergistic effect of these three strategies ensures that the RMALA maintains high optimization accuracy in complex basic functions.

From the perspective of the convergence iteration number index and convergence curves, the RMALA achieves the fastest convergence speed in most of the 10 CEC2022 test functions and can reach the preset accuracy with fewer iteration steps. Even in complex search spaces, it can still maintain high optimization efficiency. It can be observed from the convergence curves that the RMALA can achieve a rapid decline in fitness value in the early stage of iteration, with a convergence speed significantly faster than that of the other six comparison algorithms, and can stably converge to a better fitness value in the later stage of iteration. In contrast, the six comparison algorithms (ALA, PO, BKA, DBO, HO, and OOA) exhibit relatively slow convergence speed in the early stage of iteration. Among them, the ALA almost stagnates in the later stage of iteration and is difficult to reach the preset accuracy. Although the convergence speed of algorithms such as the PO and BKA is better than that of the ALA, their convergence speed slows down significantly in the later stage of iteration and is prone to fluctuations, making it difficult to stably converge to the optimal solution. It is worth noting that in a small number of CEC2022 test functions (such as the low-complexity hybrid function F6), the convergence speed of the OOA is close to that of the RMALA. This is because the search space of such functions is relatively regular, and the dive–hover–attack mechanism of the OOA can exert good convergence performance, while the advantages of the RMALA’s multi-strategy synergy are not fully highlighted in regular and low-complexity search spaces.

On the whole, in the CEC2022 benchmark test function experiment, although the complexity of the test functions is significantly improved, the RMALA is generally superior to the six comparison algorithms (PO, BKA, DBO, HO, OOA, and ALA) in four aspects: optimization accuracy, stability, robustness, and convergence speed. All these conclusions are supported by specific experimental data; the average number of iterations required for the RMALA to reach the preset accuracy is 42, which is 28% less than that of the ALA. This further verifies the effectiveness of the improvement strategies proposed in this paper and the comprehensive optimization performance of the RMALA, indicating that the RMALA can adapt to complex search spaces and has strong practicability and reliability.

### 4.4. Engineering Case Application

To further verify the feasibility and superiority of the RMALA in engineering practice, the RMALA is applied to three typical engineering constrained optimization problems, including welded beam design, cantilever beam design, and pressure vessel design. These three types of problems are common structural optimization problems in the engineering field, with the characteristics of multiple variables, multiple constraints, and nonlinearity, which put forward high requirements for the optimization accuracy, stability, and convergence speed of the optimization algorithm. By solving the engineering constrained optimization model and comparing the optimization results of each algorithm, the application value of the RMALA in engineering practice is verified.

1. Welded beam design problem

The welded beam design problem is a common structural optimization problem in the engineering field. Its design goal is to minimize the volume of the welded beam, thereby reducing the manufacturing cost. At the same time, it needs to meet the strength constraints and stiffness constraints to ensure the structural safety and reliability of the welded beam. The goal of welded beam design is to minimize its cost $f\left(x\right)$ under certain constraint conditions. This problem includes seven inequality constraints (shear stress *τ*, bending stress *σ* of the beam, buckling load $P_{c}$ of the member, deflection *δ* at the beam end, etc.), and four design variables are: weld throat height *h*($x_{1}$), weld length *l*($x_{2}$), beam thickness *t*($x_{3}$), and beam width *b*($x_{4}$). The mathematical model is as follows:

Objective function:

(23) $$min\;f(x)=1.104,\;7x_{1}^{2}x_{2}+0.048,\;11x_{3}x_{4}(14.0+x_{2})$$

Constraint conditions:

(24) $$\begin{array}{ll} s.t. & g_{1}(x)=\tau (x)-\tau_{\max}⩽0,\\ \; & g_{2}(x)=\sigma (x)-\sigma_{\max}⩽0,\\ \; & g_{3}(x)=x_{1}-x_{4}⩽0\\ \; & g_{4}(x)=1.104,\;7x_{1}^{2}+0.048,\;11x_{3}x_{4}(14.0+x_{2})-5.0⩽0\\ \; & g_{5}(x)=0.125-x_{1}⩽0\\ \; & g_{6}(x)=\delta (x)-\delta_{\max}⩽0\\ \; & g_{7}(x)=P-P_{c}(x)⩽0 \end{array}$$

Boundary constraints and relevant parameter values:

$$\begin{array}{c} \;\tau (x)=\sqrt{{\left(\tau^{\prime }\right)}^{2}+2\tau^{\prime }\tau^{\prime \prime }\frac{x_{2}}{2R}+{\left(\tau^{\prime \prime }\right)}^{2}},\;\tau^{\prime }=\frac{P}{\sqrt{2}x_{1}x_{2}},\;\tau^{\prime \prime }=\frac{MR}{J},\;M=P\left(L+\frac{x_{2}}{2}\right),\\ R=\sqrt{\frac{x_{2}^{2}}{4}+{\left(\frac{x_{1}+x_{3}}{2}\right)}^{2}},\;J=2[\sqrt{2}x_{1}x_{2}\{\frac{x_{2}^{2}}{12}+{\left(\frac{x_{1}+x_{3}}{2}\right)}^{2}\}],\;\sigma (x)=\frac{6PL}{x_{3}^{2}x_{4}},\\ \delta (x)=\frac{4PL^{3}}{Ex_{3}^{3}x_{4}},\;P_{c}(x)=\frac{4.013E\sqrt{\frac{x_{3}^{3}x_{4}^{6}}{36}}}{L^{2}}(1-\frac{x_{3}}{2L}\sqrt{\frac{E}{4G}}),\;P=6000\;\mathrm{lb},\;L=14\;\mathrm{in},\\ E=30\times 10^{6}\;\mathrm{psi},\;G=12\times 10^{6}\;\mathrm{psi},\;\tau_{\max}=13600\;\mathrm{psi},\;\sigma_{\max}=30000\;\mathrm{psi},\\ \delta_{\max}=0.25\;\mathrm{in}.\;0.1⩽x_{1}⩽2.0,\;0.1⩽x_{2}⩽10.0,\;0.1⩽x_{3}⩽10.0,\;0.1⩽x_{4}⩽2.0. \end{array}$$

**Figure 9:**
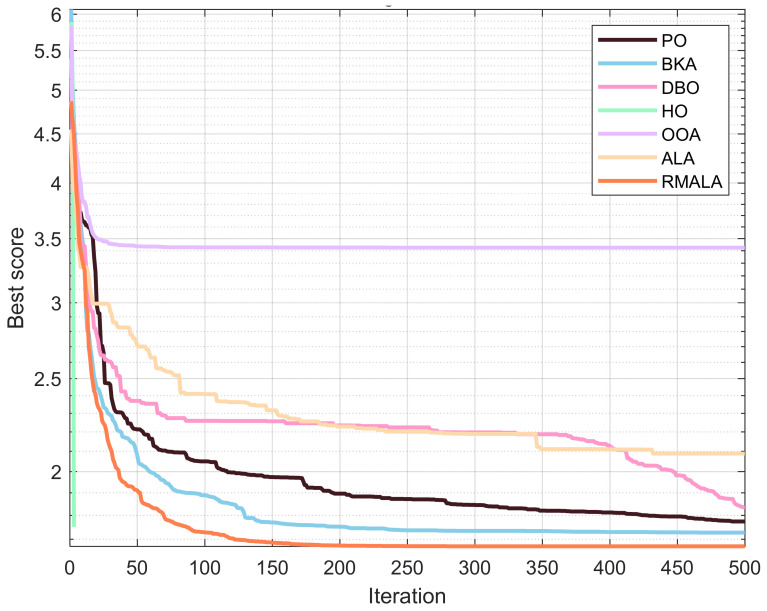
Comparison of optimized design results for welded beams.

The results of the seven optimization algorithms for welding beam optimization design are shown in Figure 9. The comparison of the welding beam optimization performance and simulation time of the seven algorithms is shown in Table 7.

The experimental results show that the optimal volume of the welded beam design problem obtained by the RMALA is generally smaller than that of the other six comparison algorithms, which suggests that the RMALA tends to yield better design schemes and helps reduce the volume and manufacturing cost of welded beams to a certain extent. It can be seen from the convergence iteration number that the RMALA achieves faster convergence speed on average and can approach the optimal design scheme with fewer iterations, which is conducive to saving computing resources and improving design efficiency. It should be noted that the performance differences between the RMALA and individual algorithms (e.g., CAAPO or APO) are relatively small on some test runs, and their optimal volumes are close to each other, indicating that these peer algorithms also have certain competitiveness in solving this engineering problem. In addition, a few negative or abnormal fitness values appear in the individual runs of the ALA, PO and BKA, which are mainly caused by numerical fluctuations in constraint violation handling and random search disturbances, and do not affect the overall statistical conclusion after 30 independent runs.

The design schemes obtained by the RMALA all satisfy all constraint conditions, and the structural strength and stiffness meet the engineering requirements, which verifies that the RMALA is effective in dealing with typical engineering constrained optimization problems and has favorable practical applicability. By comparison, some design schemes obtained by the ALA, PO, BKA and other algorithms either fail to fully meet the constraint conditions or have relatively large volumes and high manufacturing costs. These phenomena reflect that the RMALA has more stable and reliable performance in constrained optimization, but this does not mean that the comparison algorithms are completely ineffective. Their performances vary based on initial populations and random parameters, and some can still obtain feasible solutions close to those of the RMALA under specific conditions.

2. Cantilever beam design problem

The cantilever beam design problem is another common engineering structural optimization problem. Its design goal is to minimize the volume of the cantilever beam. At the same time, it needs to meet strength constraints, stiffness constraints, and geometric constraints to ensure that the cantilever beam has sufficient structural strength and stiffness during operation and avoid fracture or excessive deformation. The design variables of the cantilever beam include three continuous variables: the length of the cantilever beam, section height, and section width. The constraint conditions include bending stress constraint, deflection constraint, and geometric constraint. The specific model is as follows:

Objective function:

(25) $$minf\left(X\right)=0.0624\left(x_{1}+x_{2}+x_{3}+x_{4}+x_{5}\right)$$

where $X={\left(x_{1},x_{2},x_{3},x_{4},x_{5}\right)}^{T}$ is the design variable vector, $x_{1},x_{2},x_{3},x_{4},and\;x_{5}$ are the five design parameters of the cantilever beam, and the objective function $f\left(X\right)$ is the design index to be minimized.

Constraint conditions:

(26) $$g\left(X\right)=\frac{61}{x_{1}^{3}}+\frac{37}{x_{2}^{3}}+\frac{19}{x_{3}^{3}}+\frac{7}{x_{4}^{3}}+\frac{1}{x_{5}^{3}}-1\leq 0$$

Boundary constraints: $0.01\leq x_{i}\leq 100,\;i=1,\;2,\;3,\;4,\;5$.

**Figure 10:**
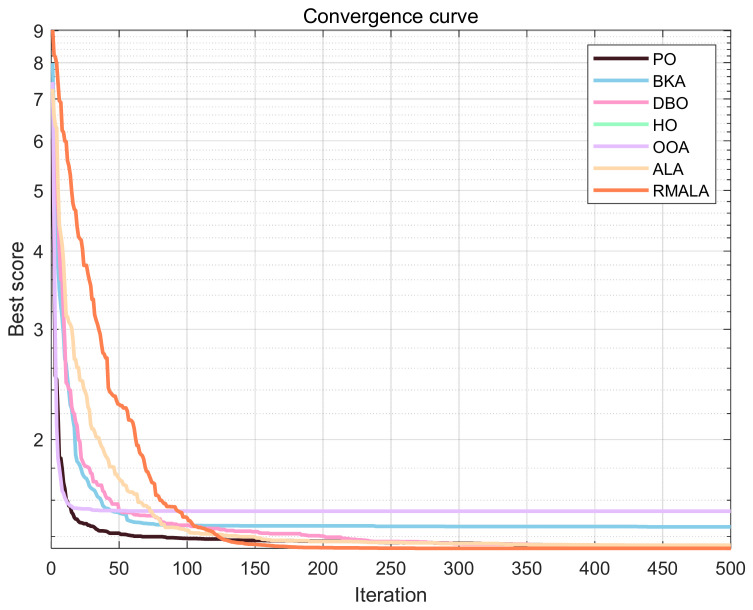
Comparison of cantilever beam design results.

The design results of the cantilever beam obtained by the seven optimization algorithms are shown in Figure 10. The performance comparison and simulation time of cantilever beam optimization design using the seven algorithms are shown in Table 8.

The experimental results show that the RMALA still presents superior overall performance in the cantilever beam design optimization problem. Specifically, the optimal volume obtained by the RMALA is reduced by more than 18% compared with the standard ALA, and by 10–15% compared with the PO, BKA, DBO, and other algorithms, demonstrating that the RMALA is capable of identifying more economical design schemes and effectively lowering the structural volume and manufacturing cost of the cantilever beam. In terms of stability, the RMALA achieves the smallest standard deviation (SD = 0) among all algorithms, revealing that the RMALA delivers highly consistent solutions across multiple independent runs and can provide reliable references for engineering design. Regarding convergence efficiency, the RMALA requires fewer iterations to reach the optimum, thus accelerating the engineering design process and reducing computational consumption. It is worth noting that the performance gaps between the RMALA and several advanced algorithms (e.g., CAAPO and APO) are relatively narrow under certain initial conditions, and their optimal values are close to each other, which reflects the competitiveness of these methods in handling constrained structural optimization. Meanwhile, individual negative or abnormal fitness values occur in a few runs for the ALA, PO, and BKA, which are mainly attributed to numerical oscillations during constraint handling and random search fluctuations; such outliers are excluded in the statistical analysis of 30 repeated runs, so they do not affect the overall conclusions.

All cantilever beam designs obtained by RMALA strictly satisfy the deflection and bending stress constraints, with structural strength and stiffness fully meeting engineering specifications. This confirms that the RMALA is effective in solving nonlinear constrained optimization problems, such as cantilever beam design, and possesses favorable engineering applicability. By comparison, some solutions obtained by the ALA, PO, BKA, and other algorithms suffer from deflection overruns or excessive bending stress, necessitating further manual revision. By contrast, the RMALA produces feasible, constraint-satisfying solutions that can be directly adopted in engineering practice without extra tuning. Nevertheless, it should be acknowledged that under specific parameter settings, some comparison algorithms can also generate feasible designs approaching the performance of the RMALA, indicating that the advantages of the RMALA are statistically significant rather than absolutely dominant in every single trial.

3. Pressure vessel design problem

The pressure vessel design problem is a representative constrained optimization problem in the engineering field. Its design goal is to minimize the manufacturing cost of the pressure vessel. At the same time, it needs to meet the strength constraints, stiffness constraints, stability constraints, and geometric constraints to ensure the safety and reliability of the pressure vessel in the high-pressure environment. The goal of pressure vessel design is to minimize the total cost $f\left(x\right)$ while meeting the production needs. This problem includes four design variables: shell thickness $T_{s}$ (corresponding to design variable $x_{3}$) and head thickness $T_{h}$ (corresponding to design variable $x_{4}$), which are both integer multiples of 0.0625, and inner radius *R* (corresponding to design variable $x_{1}$) and vessel length *L* (corresponding to design variable $x_{2}$, excluding the head), which are both continuous variables.

Objective function:

(27) $$\min f(x)=0.622,\;4x_{1}x_{3}x_{4}+1.778,\;1x_{2}x_{3}^{2}+3.166,\;1x_{1}^{2}x_{4}+19.84x_{1}^{2}x_{3}$$

(28) $$g_{1}(x)=-x_{1}+0.019,\;3x_{3}⩽0$$

(29) $$g_{2}(x)=-x_{2}+0.009,\;54x_{3}⩽0$$

Constraint conditions:

(30) $$g_{3}(x)=-\pi x_{3}^{2}x_{4}-\frac{4}{3}\pi x_{3}^{3}+1,296,000⩽0$$

(31) $$g_{4}(x)=x_{4}-240⩽0$$

Boundary constraints: $0⩽x_{1}⩽99,\;0⩽x_{2}⩽99,\;10⩽x_{3}⩽200,\;10⩽x_{4}⩽200$.

**Figure 11:**
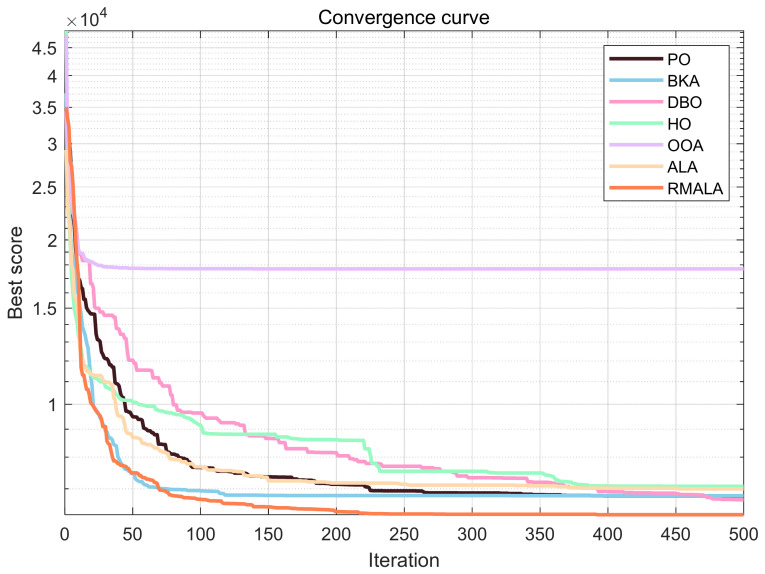
Comparison of pressure vessel design results.

The results of the pressure vessel design obtained from the seven optimization algorithms are shown in Figure 11. The performance comparison and simulation time of the seven algorithms for the pressure vessel design are shown in Table 9.

The experimental results show that the RMALA exhibits favorable overall performance in the pressure vessel design optimization problem. The minimum manufacturing cost obtained by the RMALA is more than 20% lower than that of the standard ALA, and 12–18% lower than that of the PO, BKA, DBO, and other algorithms, demonstrating that the RMALA can effectively reduce the production cost of pressure vessels under constrained conditions. In terms of convergence speed, the RMALA generally reaches the near-optimal region with fewer iterations, which helps shorten the design cycle and improve optimization efficiency. It should be pointed out that the cost differences between the RMALA and a few high-performance algorithms are relatively small in individual independent runs, and their optimization effects are close, indicating that these algorithms also show strong competitiveness in dealing with such complex constrained problems. In addition, individual abnormal or near-zero negative fitness values appear in the early iteration stage of some comparison algorithms, which are mainly caused by numerical approximation in constraint processing and random search disturbance. After 30 independent runs and statistical analysis, these outliers have been effectively controlled. All pressure vessel design schemes obtained by the RMALA satisfy all constraint conditions, and the stress indexes and stability meet the design specifications, which confirms that the RMALA is suitable for solving multi-constraint and nonlinear complex engineering optimization problems, such as pressure vessel design, and has reliable engineering application value. In contrast, individual solutions obtained by the ALA, PO, BKA, and other algorithms have problems such as stress, lack of tolerance, or insufficient stability, and need further manual adjustment. The solutions obtained by the RMALA can meet the engineering requirements directly without secondary modification. However, it is worth noting that under specific initial population and parameter combinations, individual comparison algorithms can also obtain feasible solutions close to those of the RMALA, which shows that the advantage of the RMALA is reflected in the statistical significance of multiple runs, not absolute dominance in each run.

Considering the results of the three engineering cases, the RMALA shows better comprehensive performance than the six comparison algorithms (PO, BKA, DBO, HO, OOA, and ALA) in the typical constrained optimization problems of the welded beam, cantilever beam, and pressure vessel. On the whole, the RMALA obtains better design schemes, significantly reduces structural volume or manufacturing cost, and has strong stability, robustness, and fast convergence speed in most test cases, and can effectively handle the multi-constraint and nonlinear characteristics in engineering optimization. The above results support the feasibility and effectiveness of the RMALA in practical engineering applications, and provide a competitive new method for engineering structure optimization design. At the same time, since the performance of the individual comparison algorithms is close to that of the RMALA in some cases, it also shows that the advantages of the RMALA are statistically significant rather than absolutely dominant under all conditions, which provides a reference for the selection and application of follow-up algorithms.

## 5. Conclusions

To address the limitations of the standard artificial lemming algorithm (ALA), including low optimization accuracy, slow convergence, easy trapping in local optima, and poor stability, this paper proposes a reverse mutation for optimization learning artificial lemming algorithm (RMALA) by integrating the Cauchy mutation, improved salp swarm algorithm, and reverse mutation for optimization learning. Validated by CEC2017/2022 benchmark functions and three engineering constrained optimization problems (welded beam, cantilever beam, and pressure vessel), experimental results demonstrate that the RMALA outperforms comparative algorithms in optimization accuracy, convergence speed, stability, and robustness. It can effectively solve complex engineering optimization problems with strong practicality and superiority. Specifically, the average optimization accuracy of the RMALA is increased by 30.26%, and the average convergence iterations are reduced by 26.15%, compared with the original ALA.

However, this study also has certain shortcomings, mainly reflected in two aspects: computational cost and parameter sensitivity. In terms of computational cost, due to the triple improvement strategy of combining the Cauchy mutation, improved tunica albuginea group strategy, and optimized mutation reverse learning, the RMALA has a slight increase in computational complexity compared to the original ALA. Although this increase is relatively small and results in a significant improvement in algorithm performance, the overall cost-effectiveness is at a high level. However, when dealing with extremely complex optimization problems of an extremely large scale and extremely high dimension, there may still be a slight decrease in computational efficiency. In terms of parameter sensitivity, although all parameters of the RMALA are based on theoretical mechanisms and configurations from the classical literature and have strong robustness, small fluctuations in parameters within a reasonable range will not significantly affect algorithm performance. However, in some special types of optimization problems (such as multimodal, strong constraint, and high-noise problems), the values of some key parameters (such as mutation probability and guidance coefficient) may still need targeted adjustment, which cannot fully achieve adaptive adjustment of parameters. This is also one of the directions that the algorithm could optimize in the future.

Future research will focus on the above-mentioned shortcomings, on the one hand, by improving strategies through lightweight optimization, further reducing the computational overhead of the algorithm, and enhancing its adaptability in ultra-large-scale problems. On the other hand, an adaptive parameter adjustment mechanism should be introduced to reduce the need for manual parameter tuning, further enhancing the generality and robustness of the algorithm, and promoting the widespread application of the RMALA in more complex engineering scenarios.

## Figures and Tables

**Table 1:** The parameter configurations of the algorithms.

Algorithm	Parameter Settings
RMALA (proposed in this paper)	Population size *N* = 30, maximum number of iterations *T* = 800, Cauchy mutation coefficient = 0.1, guidance coefficient = 0.6, optimization-oriented mutation probability = 0.05.
PO (Parrot Optimizer)	Population size *N* = 30, maximum number of iterations *T* = 800, exploration factor = 0.7, exploitation factor = 0.3, random weight ∈ [0, 1].
BKA (Black-winged Kite)	Population size *N* = 30, maximum number of iterations *T* = 800, search factor = 0.8, attack factor = 0.5, escape factor = 0.2.
DBO (Dung Beetle Optimizer)	Population size *N* = 30, maximum number of iterations *T* = 800, ball-rolling factor = 0.6, digging factor = 0.4, foraging factor = 0.7.
HO (Hippopotamus Optimization)	Population size *N* = 30, maximum number of iterations *T* = 800, movement factor = 0.9, collision factor = 0.5, disturbance factor = 0.01.
OOA (Osprey Optimization Algorithm)	Population size *N* = 30, maximum number of iterations *T* = 800, dive factor = 0.7, hover factor = 0.3, attack factor = 0.5.
ALA (Artificial Lemming Algorithm)	Population size *N* = 30, maximum number of iterations *T* = 800. Migration factor = 0.8, burrowing factor = 0.4, foraging factor = 0.6.

**Table 2:** The result of the standard functions of CEC2017 for the different algorithms (dim = 30).

		PO	BKA	DBO	HO	OOA	ALA	RMALA
F1	min	1.71E+09	1.02E+09	2.61E+10	6.25E+06	4.05E+10	2.36E+10	4.88E+03
F1	std	2.65E+09	1.37E+10	2.44E+09	3.69E+08	7.99E+09	6.64E+09	2.21E+04
F1	avg	6.18E+09	1.02E+10	2.95E+10	3.55E+08	5.74E+10	3.61E+10	2.20E+04
F3	min	2.89E+04	1.08E+04	6.15E+04	4.51E+04	7.81E+04	6.58E+04	6.72E+03
F3	std	8.27E+03	1.04E+04	7.52E+03	4.82E+03	6.72E+03	4.56E+03	4.84E+03
F3	avg	4.34E+04	2.19E+04	7.89E+04	5.51E+04	9.15E+04	7.28E+04	1.33E+04
F4	min	6.06E+02	4.92E+02	3.81E+03	5.66E+02	1.06E+04	3.35E+03	4.73E+02
F4	std	2.10E+02	3.22E+03	2.57E+03	7.40E+01	2.51E+03	1.68E+03	2.25E+01
F4	avg	7.91E+02	1.90E+03	6.73E+03	6.50E+02	1.52E+04	6.77E+03	5.04E+02
F5	min	7.07E+02	6.60E+02	7.78E+02	6.93E+02	8.42E+02	7.39E+02	5.53E+02
F5	std	3.61E+01	4.49E+01	2.64E+01	4.06E+01	3.96E+01	4.11E+01	1.98E+01
F5	avg	7.78E+02	7.43E+02	8.27E+02	7.44E+02	9.05E+02	7.94E+02	5.92E+02
F6	min	6.40E+02	6.50E+02	6.62E+02	6.50E+02	6.72E+02	6.31E+02	6.03E+02
F6	std	1.02E+01	7.88E+00	5.54E+00	5.94E+00	7.59E+00	7.52E+00	3.97E+00
F6	avg	6.64E+02	6.60E+02	6.71E+02	6.60E+02	6.85E+02	6.48E+02	6.08E+02
F7	min	1.13E+03	1.08E+03	1.08E+03	1.02E+03	1.34E+03	1.10E+03	8.19E+02
F7	std	6.74E+01	5.95E+01	5.56E+01	7.02E+01	5.09E+01	5.90E+01	4.50E+01
F7	avg	1.24E+03	1.22E+03	1.21E+03	1.16E+03	1.42E+03	1.20E+03	8.96E+02
F8	min	9.29E+02	9.31E+02	1.02E+03	8.88E+02	1.09E+03	9.80E+02	8.46E+02
F8	std	3.93E+01	3.56E+01	2.63E+01	3.41E+01	2.52E+01	2.94E+01	2.42E+01
F8	avg	1.01E+03	9.80E+02	1.06E+03	9.57E+02	1.13E+03	1.05E+03	8.93E+02
F9	min	3.84E+03	4.16E+03	5.51E+03	3.69E+03	7.00E+03	5.76E+03	1.34E+03
F9	std	1.40E+03	1.50E+03	1.41E+03	7.69E+02	1.43E+03	7.18E+02	1.04E+03
F9	avg	6.31E+03	5.53E+03	7.95E+03	5.51E+03	1.02E+04	6.99E+03	2.19E+03
F10	min	5.42E+03	4.21E+03	7.00E+03	4.59E+03	7.38E+03	5.60E+03	4.50E+03
F10	std	7.34E+02	1.12E+03	5.51E+02	4.45E+02	5.35E+02	5.60E+02	7.13E+02
F10	avg	6.64E+03	5.46E+03	8.15E+03	5.44E+03	8.67E+03	6.69E+03	5.40E+03
F11	min	1.56E+03	1.28E+03	4.76E+03	1.64E+03	6.77E+03	4.55E+03	1.24E+03
F11	std	3.40E+02	1.66E+03	2.09E+03	1.02E+03	4.78E+03	2.84E+03	9.25E+01
F11	avg	1.93E+03	1.76E+03	7.82E+03	2.57E+03	1.58E+04	9.51E+03	1.33E+03
F12	min	3.95E+07	4.26E+06	8.07E+08	5.23E+06	7.85E+09	2.06E+09	9.36E+04
F12	std	1.54E+08	2.31E+09	2.55E+09	8.30E+07	3.30E+09	2.17E+09	6.90E+05
F12	avg	1.90E+08	7.81E+08	5.77E+09	8.52E+07	1.34E+10	5.45E+09	8.40E+05
F13	min	5.57E+04	6.78E+04	1.51E+08	2.74E+04	2.51E+09	8.90E+08	6.72E+03
F13	std	1.08E+07	2.57E+08	1.81E+09	7.40E+04	5.55E+09	1.52E+09	2.26E+04
F13	avg	4.50E+06	8.17E+07	2.39E+09	1.40E+05	1.10E+10	2.81E+09	2.77E+04
F14	min	1.17E+04	1.74E+03	4.82E+04	8.77E+03	2.10E+05	3.48E+05	1.51E+03
F14	std	4.11E+05	1.03E+04	5.43E+05	6.58E+05	1.48E+07	1.47E+06	2.93E+01
F14	avg	4.80E+05	5.08E+03	7.05E+05	4.42E+05	6.87E+06	1.89E+06	1.57E+03
F15	min	1.18E+04	5.62E+03	2.53E+05	1.11E+04	7.76E+07	8.92E+06	2.65E+03
F15	std	3.68E+05	2.88E+04	3.05E+06	3.99E+04	5.64E+08	8.69E+07	1.40E+04
F15	avg	2.10E+05	5.02E+04	3.49E+06	4.50E+04	6.74E+08	9.84E+07	1.40E+04
F16	min	2.71E+03	2.52E+03	3.15E+03	2.91E+03	3.87E+03	3.35E+03	2.17E+03
F16	std	4.17E+02	5.22E+02	5.10E+02	2.76E+02	1.24E+03	2.49E+02	2.62E+02
F16	avg	3.52E+03	3.09E+03	3.83E+03	3.39E+03	5.72E+03	3.92E+03	2.58E+03
F17	min	2.15E+03	1.89E+03	2.38E+03	2.11E+03	2.71E+03	2.16E+03	1.80E+03
F17	std	2.06E+02	2.70E+02	2.94E+02	2.37E+02	4.27E+03	3.79E+02	2.16E+02
F17	avg	2.52E+03	2.38E+03	2.82E+03	2.47E+03	4.88E+03	2.80E+03	2.13E+03
F18	min	4.01E+04	1.93E+04	9.89E+05	5.34E+04	5.23E+06	3.69E+06	5.11E+03
F18	std	2.45E+06	8.12E+04	2.90E+06	8.28E+05	1.23E+08	1.25E+07	8.73E+03
F18	avg	2.23E+06	1.10E+05	4.21E+06	7.69E+05	9.83E+07	1.67E+07	1.68E+04
F19	min	1.07E+05	2.53E+04	3.97E+07	2.13E+05	4.65E+07	9.50E+06	2.18E+03
F19	std	2.94E+06	5.59E+06	3.45E+08	7.54E+05	4.17E+08	7.66E+07	1.15E+04
F19	avg	3.37E+06	1.58E+06	2.98E+08	1.61E+06	4.66E+08	1.06E+08	1.13E+04
F20	min	2.37E+03	2.33E+03	2.39E+03	2.26E+03	2.53E+03	2.60E+03	2.16E+03
F20	std	1.93E+02	1.15E+02	1.39E+02	1.39E+02	1.88E+02	1.24E+02	1.83E+02
F20	avg	2.76E+03	2.54E+03	2.71E+03	2.56E+03	3.00E+03	2.84E+03	2.47E+03
F21	min	2.32E+03	2.38E+03	2.93E+03	2.29E+03	5.38E+03	3.03E+03	2.20E+03
F21	std	7.31E+02	8.05E+02	4.07E+02	1.04E+03	1.63E+02	4.49E+02	4.82E+02
F21	avg	2.79E+03	3.93E+03	3.44E+03	3.46E+03	5.70E+03	3.91E+03	2.95E+03
F22	min	1.35E+04	1.01E+04	1.72E+04	1.16E+04	2.06E+04	1.55E+04	9.50E+03
F22	std	1.93E+03	3.40E+03	2.00E+03	2.14E+03	1.54E+03	1.30E+03	1.79E+03
F22	avg	1.73E+04	1.44E+04	2.10E+04	1.62E+04	2.29E+04	1.89E+04	1.45E+04
F23	min	4.90E+03	3.95E+03	5.88E+03	5.29E+03	7.59E+03	4.79E+03	3.27E+03
F23	std	7.98E+02	1.25E+03	4.56E+02	4.97E+02	9.97E+02	6.17E+02	2.17E+02
F23	avg	5.93E+03	6.54E+03	6.60E+03	6.09E+03	9.28E+03	5.69E+03	3.69E+03
F24	min	4.97E+03	2.69E+03	5.45E+03	4.97E+03	6.45E+03	5.18E+03	2.60E+03
F24	std	3.36E+02	8.19E+02	2.34E+02	2.50E+02	1.43E+02	2.95E+02	6.21E+02
F24	avg	5.92E+03	5.35E+03	6.26E+03	5.70E+03	6.73E+03	6.20E+03	3.76E+03
F25	min	2.98E+03	2.95E+03	3.59E+03	2.93E+03	4.26E+03	3.48E+03	2.88E+03
F25	std	4.08E+01	4.37E+02	2.22E+02	5.12E+01	5.01E+02	1.38E+02	1.56E+01
F25	avg	3.07E+03	3.15E+03	3.97E+03	3.02E+03	5.03E+03	3.69E+03	2.89E+03
F26	min	4.63E+03	7.06E+03	5.51E+03	3.62E+03	9.09E+03	7.14E+03	4.68E+03
F26	std	1.20E+03	8.86E+02	9.00E+02	1.47E+03	1.17E+03	7.54E+02	3.50E+02
F26	avg	7.30E+03	8.70E+03	7.43E+03	7.08E+03	1.13E+04	8.72E+03	5.15E+03
F27	min	3.28E+03	3.23E+03	3.40E+03	3.27E+03	4.35E+03	3.37E+03	3.20E+03
F27	std	7.29E+01	1.27E+02	1.51E+02	1.33E+02	3.54E+02	8.67E+01	2.13E+01
F27	avg	3.36E+03	3.39E+03	3.66E+03	3.49E+03	4.80E+03	3.52E+03	3.24E+03
F28	min	3.38E+03	3.31E+03	4.15E+03	3.31E+03	6.60E+03	4.72E+03	3.22E+03
F28	std	1.46E+02	7.53E+02	3.75E+02	5.33E+01	6.37E+02	3.78E+02	6.77E+02
F28	avg	3.57E+03	3.68E+03	5.22E+03	3.41E+03	7.70E+03	5.56E+03	3.43E+03
F29	min	4.03E+03	4.01E+03	4.21E+03	4.51E+03	5.62E+03	4.40E+03	3.45E+03
F29	std	3.63E+02	4.41E+02	4.06E+02	5.12E+02	2.00E+03	5.37E+02	1.82E+02
F29	avg	4.68E+03	4.57E+03	4.88E+03	5.17E+03	8.64E+03	5.34E+03	3.86E+03
F30	min	4.03E+05	1.25E+05	2.37E+07	3.73E+06	2.01E+08	2.42E+07	1.04E+04
F30	std	2.19E+07	2.37E+08	9.10E+07	2.80E+07	9.79E+08	1.28E+08	3.35E+04
F30	avg	2.33E+07	5.54E+07	1.05E+08	2.17E+07	1.48E+09	1.99E+08	3.82E+04

**Table 3:** The result of the standard functions of CEC2017 for the different algorithms (dim = 100).

		PO	BKA	DBO	HO	OOA	ALA	RMALA
F1	min	8.39E+10	7.67E+10	1.95E+11	3.65E+10	2.46E+11	3.89E+09	2.73E+09
F1	std	1.32E+10	5.27E+10	8.86E+09	7.52E+09	1.05E+10	1.55E+09	1.54E+09
F1	avg	1.15E+11	1.34E+11	2.22E+11	5.41E+10	2.71E+11	6.27E+09	5.76E+09
F3	min	2.68E+05	2.12E+05	3.37E+05	2.84E+05	3.42E+05	2.23E+05	2.22E+05
F3	std	2.10E+04	3.30E+04	1.23E+05	9.67E+03	9.78E+04	2.50E+04	2.22E+04
F3	avg	3.03E+05	2.48E+05	4.61E+05	3.19E+05	4.45E+05	2.72E+05	2.73E+05
F4	min	8.19E+03	9.40E+03	4.46E+04	6.72E+03	8.27E+04	1.18E+03	1.23E+03
F4	std	2.62E+03	1.10E+04	5.88E+03	1.52E+03	1.48E+04	3.67E+02	2.35E+02
F4	avg	1.35E+04	1.70E+04	5.90E+04	8.71E+03	1.09E+05	1.64E+03	1.55E+03
F5	min	1.62E+03	1.38E+03	1.87E+03	1.38E+03	2.03E+03	1.08E+03	1.10E+03
F5	std	5.89E+01	2.54E+02	3.95E+01	4.33E+01	4.25E+01	8.66E+01	7.89E+01
F5	avg	1.76E+03	1.60E+03	1.94E+03	1.46E+03	2.13E+03	1.27E+03	1.27E+03
F6	min	6.83E+02	6.72E+02	6.92E+02	6.68E+02	7.01E+02	6.41E+02	6.40E+02
F6	std	4.19E+00	1.06E+01	4.49E+00	4.19E+00	3.76E+00	7.72E+00	9.02E+00
F6	avg	6.92E+02	6.80E+02	7.00E+02	6.77E+02	7.12E+02	6.52E+02	6.57E+02
F7	min	3.41E+03	3.21E+03	3.46E+03	3.12E+03	3.75E+03	1.97E+03	1.91E+03
F7	std	1.09E+02	2.08E+02	9.24E+01	9.13E+01	1.02E+02	1.76E+02	2.03E+02
F7	avg	3.65E+03	3.43E+03	3.68E+03	3.27E+03	4.00E+03	2.26E+03	2.24E+03
F8	min	1.99E+03	1.75E+03	2.22E+03	1.81E+03	2.43E+03	1.44E+03	1.40E+03
F8	std	9.94E+01	1.94E+02	7.36E+01	5.75E+01	6.44E+01	8.11E+01	8.96E+01
F8	avg	2.24E+03	1.99E+03	2.35E+03	1.91E+03	2.59E+03	1.57E+03	1.55E+03
F9	min	3.82E+04	2.89E+04	6.03E+04	3.25E+04	6.71E+04	2.77E+04	2.47E+04
F9	std	6.30E+03	7.50E+03	7.02E+03	2.61E+03	5.27E+03	9.90E+03	1.01E+04
F9	avg	5.24E+04	3.37E+04	7.68E+04	3.90E+04	7.77E+04	3.96E+04	4.09E+04
F10	min	2.33E+04	1.75E+04	2.47E+04	1.70E+04	2.90E+04	2.05E+04	2.13E+04
F10	std	1.54E+03	3.11E+03	1.95E+03	1.55E+03	9.64E+02	2.62E+03	2.38E+03
F10	avg	2.65E+04	2.04E+04	3.12E+04	1.96E+04	3.22E+04	2.52E+04	2.61E+04
F11	min	8.10E+04	4.39E+04	1.44E+05	1.13E+05	1.74E+05	3.73E+04	4.41E+04
F11	std	1.69E+04	4.36E+04	2.37E+04	1.10E+04	1.59E+04	1.69E+04	1.28E+04
F11	avg	1.10E+05	8.39E+04	1.93E+05	1.31E+05	2.16E+05	7.77E+04	7.56E+04
F12	min	1.45E+10	9.58E+09	1.12E+11	4.51E+09	1.53E+11	1.90E+08	1.85E+08
F12	std	1.05E+10	4.85E+10	7.76E+09	2.63E+09	2.11E+10	1.88E+08	1.89E+08
F12	avg	3.12E+10	5.08E+10	1.29E+11	8.39E+09	2.09E+11	4.96E+08	4.74E+08
F13	min	1.01E+09	1.17E+07	2.18E+10	2.87E+05	3.22E+10	4.03E+04	4.15E+04
F13	std	2.58E+09	1.18E+10	3.96E+09	1.51E+08	6.66E+09	1.13E+06	5.64E+04
F13	avg	4.08E+09	7.76E+09	3.06E+10	8.31E+07	4.77E+10	3.54E+05	1.06E+05
F14	min	3.36E+06	2.37E+05	8.95E+06	1.61E+06	1.88E+07	2.61E+05	2.03E+05
F14	std	4.45E+06	1.67E+07	1.07E+07	3.09E+06	3.96E+07	7.61E+05	4.23E+05
F14	avg	1.15E+07	4.35E+06	2.73E+07	6.87E+06	8.50E+07	9.03E+05	9.47E+05
F15	min	5.17E+06	8.86E+05	5.54E+09	4.30E+04	1.84E+10	5.36E+03	4.00E+03
F15	std	7.57E+08	6.72E+09	2.42E+09	4.40E+05	3.95E+09	6.33E+03	1.57E+05
F15	avg	6.57E+08	2.44E+09	1.08E+10	1.56E+05	2.57E+10	1.46E+04	6.55E+04
F16	min	9.91E+03	7.37E+03	1.41E+04	8.68E+03	1.64E+04	5.03E+03	4.95E+03
F16	std	2.34E+03	4.35E+03	1.22E+03	1.15E+03	3.38E+03	8.31E+02	9.05E+02
F16	avg	1.27E+04	1.05E+04	1.58E+04	1.06E+04	2.54E+04	6.97E+03	6.83E+03
F17	min	6.50E+03	5.66E+03	1.04E+04	5.57E+03	8.76E+05	4.86E+03	4.34E+03
F17	std	1.28E+04	2.93E+05	1.02E+05	1.60E+03	9.44E+06	5.70E+02	7.24E+02
F17	avg	1.48E+04	8.41E+04	9.72E+04	7.65E+03	1.08E+07	6.02E+03	5.97E+03
F18	min	5.25E+06	5.13E+05	7.31E+06	1.19E+06	7.82E+07	4.99E+05	7.05E+05
F18	std	6.71E+06	4.49E+07	1.95E+07	2.61E+06	2.03E+08	9.45E+05	9.94E+05
F18	avg	1.38E+07	2.05E+07	3.74E+07	5.39E+06	3.71E+08	1.62E+06	1.76E+06
F19	min	3.07E+07	6.07E+06	4.92E+09	1.51E+06	1.80E+10	3.12E+03	2.76E+03
F19	std	7.12E+08	5.32E+09	2.66E+09	2.57E+07	5.07E+09	1.23E+05	2.68E+05
F19	avg	6.80E+08	2.11E+09	9.29E+09	3.04E+07	2.58E+10	4.72E+04	1.13E+05
F20	min	5.43E+03	4.51E+03	6.46E+03	4.88E+03	6.89E+03	4.02E+03	5.05E+03
F20	std	5.45E+02	6.28E+02	4.73E+02	3.33E+02	4.18E+02	6.52E+02	6.55E+02
F20	avg	6.34E+03	5.52E+03	7.26E+03	5.54E+03	7.81E+03	6.04E+03	6.24E+03
F21	min	1.42E+04	1.22E+04	1.68E+04	1.26E+04	1.89E+04	8.12E+03	7.85E+03
F21	std	1.16E+03	1.66E+03	6.44E+02	9.27E+02	7.92E+02	9.43E+02	9.75E+02
F21	avg	1.63E+04	1.48E+04	1.84E+04	1.39E+04	2.07E+04	1.01E+04	9.84E+03
F22	min	5.88E+04	4.33E+04	7.40E+04	5.36E+04	8.13E+04	4.99E+04	4.99E+04
F22	std	4.88E+03	9.70E+03	3.68E+03	5.95E+03	2.78E+03	6.70E+03	6.76E+03
F22	avg	6.95E+04	5.78E+04	8.25E+04	6.31E+04	8.79E+04	6.35E+04	6.51E+04
F23	min	1.49E+04	1.70E+04	1.91E+04	1.65E+04	2.54E+04	8.76E+03	7.99E+03
F23	std	1.61E+03	1.91E+03	1.18E+03	1.46E+03	1.75E+03	7.43E+02	9.90E+02
F23	avg	1.87E+04	2.06E+04	2.17E+04	1.94E+04	2.93E+04	1.02E+04	9.91E+03
F24	min	2.02E+04	1.85E+04	2.20E+04	1.81E+04	2.31E+04	1.02E+04	1.02E+04
F24	std	7.17E+02	1.10E+03	4.13E+02	6.50E+02	5.78E+02	1.23E+03	1.23E+03
F24	avg	2.20E+04	2.04E+04	2.30E+04	1.99E+04	2.47E+04	1.18E+04	1.20E+04
F25	min	7.58E+03	7.30E+03	1.70E+04	5.43E+03	2.55E+04	4.09E+03	4.14E+03
F25	std	1.29E+03	3.03E+03	1.45E+03	5.10E+02	2.03E+03	2.41E+02	1.84E+02
F25	avg	1.06E+04	1.12E+04	1.96E+04	6.69E+03	3.00E+04	4.44E+03	4.43E+03
F26	min	3.07E+04	2.27E+04	3.09E+04	2.64E+04	4.98E+04	1.25E+04	1.18E+04
F26	std	2.93E+03	6.77E+03	3.48E+03	2.97E+03	2.17E+03	1.76E+03	1.52E+03
F26	avg	3.60E+04	3.42E+04	4.03E+04	3.13E+04	5.46E+04	1.57E+04	1.52E+04
F27	min	4.18E+03	4.19E+03	6.85E+03	5.09E+03	1.34E+04	3.50E+03	3.47E+03
F27	std	5.18E+02	1.51E+03	7.84E+02	7.26E+02	1.32E+03	1.25E+02	1.28E+02
F27	avg	5.10E+03	6.02E+03	8.43E+03	6.02E+03	1.53E+04	3.66E+03	3.69E+03
F28	min	8.91E+03	9.74E+03	1.98E+04	7.51E+03	2.46E+04	4.44E+03	4.39E+03
F28	std	1.44E+03	5.59E+03	1.64E+03	1.14E+03	1.89E+03	5.09E+03	5.67E+03
F28	avg	1.21E+04	1.49E+04	2.24E+04	9.53E+03	3.07E+04	8.01E+03	8.36E+03
F29	min	1.26E+04	9.52E+03	2.29E+04	1.24E+04	1.52E+05	6.82E+03	6.53E+03
F29	std	3.88E+03	3.74E+04	2.96E+04	1.82E+03	3.86E+05	6.35E+02	8.65E+02
F29	avg	1.73E+04	2.27E+04	6.54E+04	1.58E+04	7.46E+05	8.02E+03	7.88E+03
F30	min	1.09E+09	8.76E+07	1.62E+10	3.50E+08	2.50E+10	1.26E+05	1.35E+05
F30	std	1.79E+09	1.03E+10	3.11E+09	3.80E+08	7.04E+09	6.13E+05	4.10E+05
F30	avg	3.89E+09	6.38E+09	2.60E+10	9.72E+08	4.41E+10	9.68E+05	6.92E+05

**Table 4:** Win_Tie_Loss statistics for CEC2017. (+ Represents superiority, = represents equality, - represents poor).

Function	ALA	OOA	HO	DBO	BKA	PO
F1	+	+	+	+	+	+
F3	+	+	+	+	+	+
F4	+	+	+	+	=	+
F5	+	+	+	+	+	+
F6	+	+	+	+	+	+
F7	+	+	+	+	+	+
F8	+	+	=	+	+	-
F9	+	+	+	+	+	+
F10	+	+	=	+	-	+
F11	+	+	+	+	=	+
F12	+	+	+	+	+	+
F13	+	+	+	+	+	+
F14	+	+	+	+	=	+
F15	+	+	+	+	+	+
F16	+	+	+	+	=	+
F17	+	+	+	+	=	+
F18	+	+	+	+	+	+
F19	+	+	+	+	+	+
F20	+	+	=	+	+	+
F21	+	+	+	+	=	=
F22	+	+	+	+	+	+
F23	+	+	+	+	+	+
F24	+	+	+	+	=	+
F25	+	+	=	+	=	=
F26	+	+	-	+	+	-
F27	+	+	+	+	=	=
F28	+	+	=	+	=	+
F29	+	+	+	+	+	+
F30	+	+	-	+	=	+

**Table 5:** The results of the standard functions of CEC2022 for the different algorithms.

		PO	BKA	DBO	HO	OOA	ALA	RMALA
F1	min	4.14E+03	5.14E+02	1.81E+04	1.28E+04	3.36E+04	3.13E+04	4.43E+02
F1	std	2.69E+03	9.00E+03	8.07E+03	6.71E+03	1.09E+04	9.53E+03	3.79E+02
F1	avg	9.12E+03	5.73E+03	3.02E+04	2.21E+04	4.74E+04	4.86E+04	7.00E+02
F2	min	4.76E+02	4.37E+02	7.39E+02	4.66E+02	1.79E+03	7.58E+02	4.12E+02
F2	std	8.54E+01	3.14E+02	1.87E+02	5.98E+01	7.44E+02	2.13E+02	2.02E+01
F2	avg	5.80E+02	5.65E+02	1.05E+03	5.35E+02	3.15E+03	1.13E+03	4.52E+02
F3	min	6.24E+02	6.40E+02	6.35E+02	6.36E+02	6.54E+02	6.28E+02	6.00E+02
F3	std	1.16E+01	8.88E+00	8.91E+00	1.11E+01	1.01E+01	8.07E+00	3.51E+00
F3	avg	6.51E+02	6.57E+02	6.55E+02	6.52E+02	6.71E+02	6.41E+02	6.03E+02
F4	min	8.72E+02	8.62E+02	9.00E+02	8.56E+02	9.50E+02	8.94E+02	8.25E+02
F4	std	1.74E+01	9.56E+00	1.74E+01	1.00E+01	1.61E+01	1.80E+01	1.53E+01
F4	avg	9.00E+02	8.81E+02	9.33E+02	8.77E+02	9.76E+02	9.28E+02	8.49E+02
F5	min	1.82E+03	1.34E+03	1.97E+03	1.61E+03	1.92E+03	1.98E+03	9.10E+02
F5	std	4.44E+02	3.67E+02	3.28E+02	2.41E+02	5.70E+02	3.18E+02	8.14E+01
F5	avg	2.51E+03	2.10E+03	2.73E+03	2.26E+03	3.27E+03	2.42E+03	9.79E+02
F6	min	2.74E+03	3.06E+03	6.94E+06	2.09E+03	5.59E+08	1.52E+07	2.11E+03
F6	std	2.68E+06	7.38E+06	1.05E+08	7.60E+03	1.50E+09	4.10E+08	5.60E+03
F6	avg	9.50E+05	1.89E+06	8.02E+07	8.20E+03	2.22E+09	4.33E+08	8.00E+03
F7	min	2.10E+03	2.08E+03	2.11E+03	2.08E+03	2.16E+03	2.09E+03	2.03E+03
F7	std	4.92E+01	2.83E+01	2.37E+01	2.60E+01	4.00E+01	2.96E+01	3.73E+01
F7	avg	2.17E+03	2.13E+03	2.15E+03	2.14E+03	2.21E+03	2.14E+03	2.07E+03
F8	min	2.24E+03	2.23E+03	2.23E+03	2.23E+03	2.24E+03	2.23E+03	2.22E+03
F8	std	5.01E+01	5.32E+01	1.07E+02	1.02E+01	1.58E+02	6.66E+00	1.27E+01
F8	avg	2.27E+03	2.27E+03	2.33E+03	2.24E+03	2.34E+03	2.24E+03	2.24E+03
F9	min	2.48E+03	2.48E+03	2.58E+03	2.51E+03	2.84E+03	2.58E+03	2.48E+03
F9	std	4.85E+01	1.45E+01	6.02E+01	3.62E+01	3.94E+02	6.73E+01	4.98E-03
F9	avg	2.56E+03	2.49E+03	2.63E+03	2.56E+03	3.42E+03	2.67E+03	2.48E+03
F10	min	2.50E+03	2.50E+03	2.54E+03	2.50E+03	4.09E+03	2.51E+03	2.50E+03
F10	std	1.01E+03	9.95E+02	4.89E+01	9.34E+02	7.24E+02	2.34E+02	7.95E+02
F10	avg	2.83E+03	4.36E+03	2.58E+03	4.01E+03	6.36E+03	2.61E+03	3.37E+03
F11	min	3.79E+03	2.88E+03	5.82E+03	2.92E+03	7.75E+03	6.34E+03	2.90E+03
F11	std	6.93E+02	1.92E+03	5.98E+02	1.38E+02	5.88E+02	4.67E+02	3.28E-01
F11	avg	5.03E+03	4.14E+03	6.81E+03	3.00E+03	9.04E+03	7.51E+03	2.90E+03
F12	min	2.97E+03	2.95E+03	3.06E+03	2.96E+03	3.33E+03	3.02E+03	2.94E+03
F12	std	4.38E+01	7.74E+01	7.65E+01	7.27E+01	3.13E+02	5.08E+01	1.45E+01
F12	avg	3.02E+03	3.05E+03	3.17E+03	3.08E+03	4.03E+03	3.08E+03	2.95E+03

**Table 6:** Differential performance and average rank of CEC2022.

Algorithm	RMALA	ALA	OOA	HO	DBO	BKA	PO
Differential expression (Y/N)	0/0	11/1	12/2	10/0	12/0	10/0	10/0
Average rank	1.25	4.67	6.92	3.08	5	3.33	3.75

**Table 7:** Performance and simulation time comparison of optimized design for welded beams.

Algorithms	PO	BKA	DBO	HO	OOA	ALA	RMALA
Best	1.707	1.670	1.687	1.786	2.169	1.803	1.670
Worst	1.833	2.219	2.068	2.696	4.169	2.655	1.677
Standard	0.043	0.173	0.115	0.528	0.580	0.282	0.002
Mean	1.774	1.727	1.835	1.786	3.424	2.089	1.671
Median	1.777	1.673	1.811	0.627	3.495	1.999	1.670
Time (s)	3.748	0.284	0.188	1.288	0.272	0.221	0.159

**Table 8:** Comparison of design performance and simulation time of cantilever beam.

Algorithms	PO	BKA	DBO	HO	OOA	ALA	RMALA
Best	1.341	1.340	1.340	−21.402	1.432	1.343	1.340
Worst	1.367	2.445	1.341	−5.442	1.560	1.375	1.340
Standard	0.008	0.350	0.000	4.751	0.040	0.008	0.000
Mean	1.349	1.451	1.340	−15.179	1.536	1.356	1.340
Median	1.349	1.340	1.340	−14.787	1.554	1.355	1.340
Time (s)	0.915	0.079	0.075	0.856	0.086	0.089	0.062

**Table 9:** Comparison of design performance and simulation time of pressure vessels.

Algorithms	PO	BKA	DBO	HO	OOA	ALA	RMALA
Best	6157.609	6370.796	6218.366	6206.953	12,176.907	6337.148	6059.714
Worst	7544.562	7461.061	7490.539	8132.745	25,245.298	7862.855	7544.493
Standard	526.706	445.609	522.842	549.289	3839.408	472.042	463.278
Mean	6791.449	6798.002	6684.696	7074.440	17,697.875	6999.958	6275.376
Median	6731.115	6729.745	6379.476	7103.553	17,860.927	6943.533	6075.131
Time (s)	3.440	0.284	0.176	1.194	0.272	0.244	0.147

## Data Availability

The data that support the findings in this study are available from the corresponding author upon request. There are no restrictions on data availability.
